# A particle flow filter for high‐dimensional system applications

**DOI:** 10.1002/qj.4028

**Published:** 2021-05-05

**Authors:** Chih‐Chi Hu, Peter Jan van Leeuwen

**Affiliations:** ^1^ Department of Atmospheric Science Colorado State University Fort Collins Colorado USA; ^2^ Department of Meteorology University of Reading Reading UK

**Keywords:** high‐dimensional system, kernel embedding, non‐Gaussian distribution, nonlinear data assimilation, particle filters, particle flows

## Abstract

A novel particle filter proposed recently, the particle flow filter (PFF), avoids the long‐existing weight degeneracy problem in particle filters and, therefore, has great potential to be applied in high‐dimensional systems. The PFF adopts the idea of a particle flow, which sequentially pushes the particles from the prior to the posterior distribution, without changing the weight of each particle. The essence of the PFF is that it assumes the particle flow is embedded in a reproducing kernel Hilbert space, so that a practical solution for the particle flow is obtained. The particle flow is independent of the choice of kernel in the limit of an infinite number of particles. Given a finite number of particles, we have found that a scalar kernel fails in high‐dimensional and sparsely observed settings. A new matrix‐valued kernel is proposed that prevents the collapse of the marginal distribution of observed variables in a high‐dimensional system. The performance of the PFF is tested and compared with a well‐tuned local ensemble transform Kalman filter (LETKF) using the 1,000‐dimensional Lorenz 96 model. It is shown that the PFF is comparable to the LETKF for linear observations, except that explicit covariance inflation is not necessary for the PFF. For nonlinear observations, the PFF outperforms LETKF and is able to capture the multimodal likelihood behavior, demonstrating that the PFF is a viable path to fully nonlinear geophysical data assimilation.

## Graphical abstract

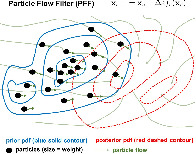

The standard particle filter suffers from a weight degeneracy problem in a high‐dimensional system. A new particle flow method, the particle flow filter (PFF), iteratively transforms the particles in space and keeps all the particles at equal weight all the time. We demonstrate that the PFF outperforms the LETKF for nonlinear observations in a high‐dimensional system.

## 1 INTRODUCTION

The advancement of numerical weather prediction depends mainly on two factors: a model that can well depict the evolution of the system, and a desirable representation of the initial condition in the model. The importance of the latter can be significant if the underlying system is chaotic, in which case a small perturbation in the initial condition can grow rapidly in a short period of time. Data assimilation is a way to improve the initial state of the system.

To be more specific, the goal of the data assimilation is to sequentially estimate the probability of each possible model state given the information of the model forecast and observations. This means that the model state vector **x** (in ${\mathbb{R}}^{n_{x}}$, where *n*_*x*_ is the dimension of the model space) is treated as a random vector. The goal is then to best describe the probability density function (pdf) of this random vector **x**, given both the model forecast and the observations. We thus need to know how the forecast, or prior, pdf is updated with the information of the observations **y**. In other words, we need to find the conditional probability of the model state given the knowledge of the observations. Using Bayes' theorem, the conditional probability *p*(**x**|**y**) can be written as

(1) $$p\left(x|y\right)=\frac{p\left(y|x\right)}{p\left(y\right)}p\left(x\right)$$

where *p*(**x**) is called the prior, *p*(**y**|**x**) is the likelihood, and *p*(**x**|**y**) is the posterior pdf.

To specify the prior, we need to evaluate the evolution of the pdf in the system. However, such evaluation of the pdf in a high‐dimensional and nonlinear system is unachievable because of the high computational cost. A typical numerical weather prediction model has over 10^9^ prognostic variables, and assimilates over 10^7^ observations every 6–12 hr (Van Leeuwen *et al*., 2019). A clever way to obtain the pdf is to adopt Monte Carlo methods. Monte Carlo methods are ways to approximate the evolution of a pdf given by the nonlinear system. First, randomly sample the initial pdf, and let the nonlinear system propagate the state of each sample. Then, use the subsequent distribution of the samples as an approximation of the prior pdf. However, it is difficult to infer the mathematical form of the pdf given a finite number of samples. An easy and useful way is to assume the pdf is Gaussian, and infer the statistics (i.e., mean and covariance) from the finite samples, as routinely done in ensemble Kalman filters (EnKF, Evensen, 1994; Houtekamer and Mitchell, 1998; Burgers *et al*., 1998; see also Van Leeuwen, 2020 for a modern interpretation of the original EnKF).

The likelihood contains the relation between model state and the observations. If the observation errors are assumed to be Gaussian, as can often be done, and if the observation operator that relates the model state to the observations is linear, then the likelihood is Gaussian. Combined with a Gaussian prior, this leads to a Gaussian posterior, and its mean and covariance can be derived analytically; see, for example, the Kalman filter. However, when the observation is nonlinearly related to the model state (for example, in the case of satellite radiance observations), the Gaussian assumption for the likelihood fails.

One way to relax the Gaussian assumption on the likelihood is to use iterative methods such as a variational method. Variational methods seek the most likely model state in the posterior pdf by directly setting the gradient of the logarithm of the posterior pdf equal to zero. However, the underlying Gauss–Newton iterations can only find the solution closest to the first guess state, often taken as the prior mean, which is not the optimal solution if the posterior is multimodal. In addition, variational methods do not provide complete error estimates of the posterior, although they can be used to obtain an approximation of the inverse of the Hessian at the local mode. Hybrid methods, which combine ideas from ensemble Kalman filters and variational methods, suffer from similar problems when the posterior pdf is multimodal.

Although the posterior pdf can often be expected to be unimodal, for example, at the large scales in atmospheric models, related to the enormous amount of observations, it is well known that it can be multimodal at smaller scales. While recent studies have shown successes for data assimilation experiments using the EnKF or hybrids with the variational method even in convective scales (e.g., Snyder and Zhang, 2003; Zhang *et al*., 2009; Jones *et al*., 2016), many of them need several ad hoc settings, such as different ways to do the localization and the inflation to tackle the inappropriate assumptions. Some studies have come up with some clever ways to adaptively deal with these ad hoc settings (e.g., Zhang *et al*., 2004; Anderson, 2007; Whitaker and Hamill, 2012; Ying and Zhang, 2015; Minamide and Zhang, 2019). Nevertheless, it is unclear whether the existing EnKF and variational methods based on the inappropriate assumptions are optimal. In other words, it is suggested that improvements on existing methods are in high demand in many geoscience data assimilation applications, and indeed possible.

One method that is not limited by any constraints in the prior distribution and linearity assumptions is the particle filter. It has the potential to better describe the full posterior pdf. However, the standard particle filter is known to suffer from the problem of weight degeneracy in high‐dimensional problems (e.g., see Snyder *et al*., 2008; Van Leeuwen, 2009 for details). This problem has made the application of particle filters in geoscience models difficult. Recently, much progress has been made to deal with the weight degeneracy problems, including the usage of the proposal density (e.g., Van Leeuwen, 2010), the introduction of localization in particle filters (e.g., Bengtsson *et al*., 2003; Van Leeuwen, 2003; Poterjoy, 2016; Poterjoy *et al*., 2019), and methods that try to transform the variables to Gaussian variables (Chorin *et al*., 2010; Morzfeld *et al*., 2012). See Van Leeuwen *et al*., 2019 for a detailed review of these methods.

The so‐called particle flow filter (Daum and Huang, 2011), a relatively new development in particle filtering, keeps all the particles at equal weight all the time. The particles are iteratively transformed from the prior to the posterior in state space, without the need to resample or to reweight the particles. Liu and Wang (2016) developed a static variant that is applicable to high‐dimensional spaces by embedding the transformation in a reproducing kernel Hilbert space. The sequential version of the Liu and Wang (2016) algorithm was developed by Pulido and van Leeuwen (2019). Pulido *et al*. (2019) successfully assimilated nonlinear observations, or observations with bimodal likelihood in the 40‐variable Lorenz 1996 system. Since the PFF avoids the weight degeneracy problem, it has the potential to be applied in a high‐dimensional system.

The motivation of this study is to investigate how to apply the PFF to high‐dimensional nonlinear problems, and to compare the performance of the PFF with the local ensemble transform Kalman filter (LETKF), which has been widely used in geoscience applications. Special emphasis will be given to the formulation of the kernel, and it is shown that the scalar kernel used in earlier versions is insufficient in high‐dimensional sparsely observed settings. A solution is found in matrix‐valued kernels. We also discuss ways to formulate the prior from the forecast particles, another important ingredient in the methodology.

The remainder of this paper is organized as follows: Section 2 introduces the theoretical background for the PFF, and the effect of the different kernels will be discussed. Section 3 describes the data assimilation experiments used to evaluate the performance of the LETKF and the PFF. Section 4 compares the experiment results between a well‐tuned LETKF and the PFF with linear and nonlinear observations. Section 5 conducts sensitivity experiments to see the effect of different settings in the PFF. Section 6 summarizes the results and discusses future applications to geosciences models.

## 2 METHODOLOGY

### 2.1 Introduction to the particle flow filter (PFF)

The particle flow filter (PFF, Pulido and van Leeuwen, 2019) iteratively transforms the particles from the prior to the posterior, with all their weights unchanged. In fact, weights play no role in this methodology. Specifically, the idea of particle flow is to (continuously) transform each state vector such that the pdf of the model state (recall that we treat the model state as a random vector) is transformed from the prior pdf to the posterior pdf. We define a pseudo time *s*, during which each state vector is transformed. We can then formulate the idea as

(2) $$\begin{array}{l} \frac{d}{\mathrm{ds}}x_{s}=f_{s}\left(x_{s}\right),\;s\in \left[0,\infty \right]\\ q_{0}\left(x\right)=p\left(x\right)\\ q_{\infty }\left(x\right)=p\left(x|y\right) \end{array}$$

where **x**_*s*_ is a state at pseudo time *s*, **f**_*s*_ is the particle flow (the transformation) that can be evaluated at each state at pseudo time *s*, and *q*_*s*_ is the intermediate pdf of the state at pseudo time *s*: in particular, *q*_0_ is the prior pdf, and *q*_∞_ is the targeted pdf, which in our case is the posterior pdf.

The flow field **f**_*s*_ has to be chosen such that the distance between the pdf at pseudo time *s* (*q*_*s*_) and the target pdf *q*_∞_(**x**) = *p*(**x**|**y**) decreases as the pseudo time increases. Here, the Kullback–Leibler divergence (KL divergence) is used as a measure of this distance. (Formally the KL divergence is not a distance measure because it is not symmetric in its two arguments, but that is not a problem here.) Specifically, the KL divergence between the intermediate pdf at pseudo time *s* and the target pdf is

(3) $$\mathrm{KL}\left(q_{s}\right)=\int q_{s}\left(x\right)\log\left(\frac{q_{s}\left(x\right)}{q_{\infty }\left(x\right)}\right)dx$$

For a given targeted pdf, and a given prior from the forecast, the KL divergence is only a function of the intermediate pdf at each pseudo time *s*. To be efficient, the aim is to find the appropriate flow field **f**_*s*_ at each pseudo time such that the KL divergence can decrease as fast as possible.

There is an infinite number of choices for the flow field. To obtain a tractable solution, **f**_*s*_ is assumed to be in a reproducing kernel Hilbert space with a kernel **K**, which is a mapping from ${\mathbb{R}}^{n_{x}}\times {\mathbb{R}}^{n_{x}}\to M_{n_{x}\times n_{x}}\left(\mathbb{R}\right)$, where *n*_*x*_ is the dimension of the system and $M_{n_{x}\times n_{x}}\left(\mathbb{R}\right)$ is a matrix of size *n*_*x*_‐by‐*n*_*x*_. By this assumption, we can derive the flow field **f**_*s*_ such that the KL divergence always decreases with respect to the pseudo time *s* (see Appendix for the derivations), as

(4) $$f_{s}\left(\cdot \right)=D\int q_{s}\left(x\right)\left\{K\left(x,\cdot \right)\nabla_{x}\log\left(p\left(x|y\right)\right)+\nabla_{x}\cdot K\left(x,\cdot \right)\right\}dx$$

where **D** is a positive‐definite matrix that we can choose. The matrix **D** is restricted in that it should ensure that the physical dimensions of **f**_*s*_ are those of the state vector, and we choose it equal to the localized prior covariance matrix. The Monte Carlo method is applied in the PFF. Denote the state of particles as $x_{s}^{1:N_{p}}$, where the superscript is the index for particles and the subscript is the pseudo time. We use the particle representation for the intermediate pdf *q*_*s*_(**x**), as shown in Equation (5):

(5) $$q_{s}\left(x\right)=\frac{1}{N_{p}}\sum_{i=1}^{N_{p}}\delta \left(x-x_{s}^{i}\right)$$

and, therefore, the flow field at pseudo time *s* in Equation (4) can be written as

(6) $$f_{s}\left(x\right)=\frac{1}{N_{p}}D\sum_{i=1}^{N_{p}}\left\{K\left(x_{s}^{i},x\right)\nabla_{x_{s}^{i}}\text{logp}\left(x_{s}^{i}|y\right)+\nabla_{x_{s}^{i}}\cdot K\left(x_{s}^{i},x\right)\right\}$$

Note that we have replaced the dot in Equation (4) with the state **x** whose particle flow is being evaluated in Equation (6). This formula is general for evaluating the transformation for any state, despite that we only need to evaluate the transformation at $x_{s}^{1:N_{p}}$ for the *N*_*p*_ particles. To implement the PFF, we can discretize Equation (2) in pseudo time, and use Equation (6) to evaluate the movement of any state at pseudo time *s* to pseudo time *s* + ∆*s*:

(7) $$\begin{array}{cc} x_{s\;+\;∆s} & =x_{s}+\frac{∆s}{N_{p}}D\sum_{i=1}^{N_{p}}\\ \left\{K\left(x_{s}^{i},x_{s}\right)\nabla_{x_{s}^{i}}\text{log p}\left(x_{s}^{i}|y\right)+\nabla_{x_{s}^{i}}\cdot K\left(x_{s}^{i},x_{s}\right)\right\} & \end{array}$$

with small ∆*s*.

**FIGURE 1:**
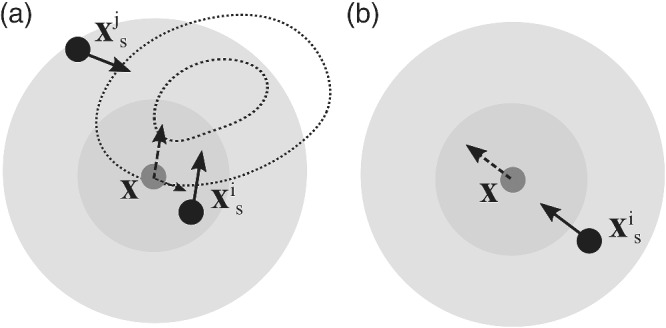
Demonstration of how the particle flow is represented by particles. The black dots are the states of particles, and the gray dot is the state **x** at which the particle flow is being evaluated. The shaded gray circles are the kernel, with darker shading representing greater value. (a) The weighted average of the gradient of the logarithm of posterior (the first term in Equation (6)). The dashed contours denote the posterior pdf. The solid arrows are the gradients at particles $x_{s}^{i}$ and $x_{s}^{j}$, which point toward the local maximum of the posterior pdf. The dashed arrow is the weighted average of the two solid arrows. Since $x_{s}^{i}$ has a larger kernel value, the particle flow evaluated at **x** has a larger portion from $x_{s}^{i}$ than from $x_{s}^{j}$. (b) The divergence from the kernel (the second term in Equation (6)). The solid arrow is the direction of the gradient of the scalar kernel evaluated at the particle $x_{s}^{i}$, while this flow is actually acting on the state **x**, which is shown by the dashed arrow

The first term on the right‐hand side (RHS) of Equation (6) is an attracting term, which drives the state toward the local maximum of the posterior pdf, as the gradient of a function points toward its local maximum value. The kernel $K\left(x_{s}^{i},x\right)$ measures how each particle $x_{s}^{1:N_{p}}$ contributes to the local particle flow of the state **x** that is being evaluated. Therefore, the kernel in the first term acts as a weighting coefficient for each $\nabla_{x_{s}^{i}}\text{logp}\left(x_{s}^{i}|y\right)$. Note that the kernel should give larger weighting to those particles that are close to **x** to get an accurate average gradient of the posterior for state **x**. In other words, the first term is the smoothed gradient at the state **x** represented by the neighboring particles. Figure 1a shows an example of how the kernel works.

The second term on the RHS of Equation (6) acts as a repelling term. To demonstrate its repelling nature, assume we have a scalar kernel:

(8) $$K\left(x_{s}^{i},x\right)=K\left(x_{s}^{i},x\right)I_{n_{x}}$$

where *K* is a function from ${\mathbb{R}}^{n_{x}}\times {\mathbb{R}}^{n_{x}}\to \mathbb{R}$, for instance:

(9) $$K\left(x_{s}^{i},x\right)=\exp\left(-\frac{1}{2}{\left(x_{s}^{i}-x\right)}^{T}A\left(x_{s}^{i}-x\right)\right)$$

Then, the divergence of the kernel is reduced to the gradient of the scalar function:

(10) $$\nabla_{x_{s}^{i}}\cdot K\left(x_{s}^{i},x\right)=\left[\begin{array}{c} \begin{array}{c} \frac{\partial }{\partial x_{1}^{i}}K\left(x_{s}^{i},x\right)\\ \frac{\partial }{\partial x_{2}^{i}}K\left(x_{s}^{i},x\right)\\ \vdots \end{array}\\ \frac{\partial }{\partial x_{n_{x}}^{i}}K\left(x_{s}^{i},x\right) \end{array}\right]=\nabla_{x_{s}^{i}}K\left(x_{s}^{i},x\right)$$

Figure 1b shows an example. The gradient of the kernel should be evaluated at the particle positions $x_{s}^{i}$ (i.e., **x** is fixed), and hence points from the particle $x_{s}^{i}$ toward the state being evaluated **x**, while this “force” is actually acting on the state being evaluated **x**. That is, the gradient of the kernel tends to separate the state being evaluated **x** from each of the particles $x_{s}^{i}$. Note that this behavior is true for any form of the kernel that maximizes when its two arguments are the same.

We can summarize the effect of each term in the particle flow as follows. Suppose that the posterior is a unimodal distribution; then the first term in the particle flow tends to make all the particles collapse into the mode, which is similar to the variational method in finding the most likely state. The second term tends to separate the particles from each other. When all the particles reach a steady state between these two forces, the distribution of the particles will follow the posterior pdf.

When evaluating Equation (6), the gradients of the logarithm of posterior (see Section 2.2) and of the kernel (see Section 2.3) need to be determined, which will be discussed in the following sections.

### 2.2 The logarithm of the posterior in the PFF

The gradient of the logarithm of the posterior can be calculated analytically if we specify the form of the prior and the likelihood. This is exactly the same as in a variational method. Specifically, based on Bayes' theorem (Equation (1)), we have

(11) $$\nabla_{x}\log p\left(x|y\right)=\nabla_{x}\log p\left(y|x\right)+\nabla_{x}\log p\left(x\right)$$

To evaluate the gradient of the logarithm of the likelihood, we could, for instance, assume a Gaussian observational error, leading to

(12) $$\log p\left(y|x\right)\propto -\frac{1}{2}{\left\|y-H\left(x\right)\right\|}_{R}$$

where ‖**y** − *H*(**x**)‖_**R**_ = (**y** − *H*(**x**))^*T*^ **R** ^−1^(**y** − *H*(**x**)), *H* is the observation operator, and **R** is the observation error covariance. Therefore, the gradient of the logarithm of likelihood can be obtained analytically,

(13) $$\nabla_{x}\log p\left(y|x\right)=H^{T}R^{-1}\left(y-H\left(x\right)\right)$$

where **H** is the linearized observation operator:

(14) $$H≔\frac{\mathrm{dH}}{dx}\left(x\right)$$

Note that the observation error distribution does not have to be Gaussian in the PFF. This is a strong point of the PFF since the observation error may be non‐Gaussian, for instance, when the observation operator *H* has a complex representation error.

When the observation operator *H* is linear, the linearized observation operator **H** is independent of the state **x**. In the EnKF, the observation operator *H* is typically evaluated at each ensemble member, after which covariance is determined between state and observation space. This covariance is not state dependent, which is not optimal if *H* is highly nonlinear. The advantage of the PFF is that it can evaluate **H** locally for each ensemble member, which gives the PFF great potential to be applied to nonlinear problems.

For the gradient of the prior, we can for instance assume the prior to be Gaussian distributed, so that the gradient can be obtained analytically as

(15) $$\nabla_{x}\log p\left(x\right)=B^{-1}\left(x-x_{b}\right)$$

where **x**_**b**_ and **B** are the prior mean and covariance matrix, respectively. Note that also the prior does not have to be the Gaussian in the PFF. Any form of pdf can be used for the prior in PFF as long as the gradient of its logarithm can be easily obtained.

### 2.3 The choice of kernel

The solution of the PFF is independent of the choice of the kernel for an infinite ensemble size (Lu *et al*., 2019). However, with only a finite number of particles, the particle distribution may not be unique. In other words, given different kernels (i.e., resulting in different particle flows), the final position of the particles in state space will be different, but their statistics should represent the posterior pdf as accurately as possible.

When the PFF was first developed, the kernel was chosen to be diagonal and isotropic, that is,

(16) $$K\left(x,z\right)=K\left(x,z\right)I_{n_{x}}$$

where *K* is a function from ${\mathbb{R}}^{n_{x}}\times {\mathbb{R}}^{n_{x}}\to \mathbb{R}$:

(17) $$K\left(x,z\right)=\exp\left(-\frac{1}{2}{\left(x-z\right)}^{T}A\left(x-z\right)\right)$$

and **A** is a matrix that can properly define the distance between particles in space. For example, we can choose **A** to be proportional to the inverse of the prior covariance:

(18) $$A={\left(\alpha B\right)}^{-1}$$

To contrast with a kernel that will be introduced later, we will refer to the kernel in Equation (16) as the scalar kernel. The divergence of the scalar kernel is

(19) $$\nabla_{x}\cdot K\left(x,z\right)=\left[\begin{array}{c} \begin{array}{c} \frac{\partial }{\partial x_{1}}K\left(x,z\right)\\ \frac{\partial }{\partial x_{2}}K\left(x,z\right)\\ \vdots \end{array}\\ \frac{\partial }{\partial x_{n_{x}}}K\left(x,z\right) \end{array}\right]=-A^{T}\left(x-z\right)K\left(x,z\right)$$

Pulido and van Leeuwen (2019) have shown that the scalar kernel works well for the 40‐variable Lorenz 96 system with 20 observations (50% of the system is observed). Lu *et al*. (2019) has shown what any symmetric smooth kernel will do when the ensemble size is infinitely large, but when the ensemble size is much smaller than the dimension of the system, care needs to be taken in formulating the kernel. When we extend the system to 1,000 variables with 250 observations (25% of the system is observed), problems arise when using the scalar kernel, no matter the specific shape of *K*.

In this study, we generalize the scalar kernel to the matrix‐valued kernel with

(20) $$K\left(x,z\right)=\mathrm{diag}\left(\left[K_{\left(1\right)}\left(x,z\right),K_{\left(2\right)}\left(x,z\right),\ldots ,K_{\left(n_{x}\right)}\left(x,z\right)\right]\right)$$

where

(21) $$K_{\left(a\right)}\left(x,z\right)=K_{\left(a\right)}\left(x_{\left(a\right)},z_{\left(a\right)}\right)=\exp\left(-\frac{1}{2}\frac{{\left(x_{\left(a\right)}-z_{\left(a\right)}\right)}^{2}}{\alpha {\sigma_{\left(a\right)}}^{2}}\right)$$

where *x*_(*a*)_ is the *a*‐th component of the vector **x**, that is,

(22) $$x=\left[\begin{array}{c} \begin{array}{c} x_{\left(1\right)}\\ x_{\left(2\right)}\\ \vdots \end{array}\\ x_{\left(n_{x}\right)} \end{array}\right]$$

and *σ*_(*a*)_ is the *SD* of the *a*‐th component for the prior, and *α* is a tunable multiplication factor determining the width of kernel, which is chosen to be the reciprocal of the number of particles. The sensitivity of the PFF to the kernel width *α* will be discussed in Section 5. The divergence of the matrix‐valued kernel is

(23) $$\begin{array}{ll} \nabla_{x_{s}^{i}}\cdot K\left(x_{s}^{i},x\right) & =\left[\begin{array}{c} \begin{array}{c} \frac{\partial }{\partial x_{1}^{i}}K_{\left(1\right)}\left(x_{s,\left(1\right)}^{i},x_{\left(1\right)}\right)\\ \frac{\partial }{\partial x_{2}^{i}}K_{\left(2\right)}\left(x_{s,\left(2\right)}^{i},x_{\left(2\right)}\right)\\ \vdots \end{array}\\ \frac{\partial }{\partial x_{n_{x}}^{i}}K_{\left(n_{x}\right)}\left(x_{s,\left(n_{x}\right)}^{i},x_{\left(n_{x}\right)}\right) \end{array}\right]\\ & =\left[\begin{array}{c} \begin{array}{c} -\left(\frac{x_{s,\left(1\right)}^{i}-x_{\left(1\right)}}{{{\alpha \sigma }_{\left(1\right)}}^{2}}\right)K_{\left(1\right)}\left(x_{s,\left(1\right)}^{i},x_{\left(1\right)}\right)\\ -\left(\frac{x_{s,\left(2\right)}^{i}-x_{\left(2\right)}}{{{\alpha \sigma }_{\left(2\right)}}^{2}}\right)K_{\left(2\right)}\left(x_{s,\left(2\right)}^{i},x_{\left(2\right)}\right)\\ \vdots \end{array}\\ -\left(\frac{x_{s,\left(n_{x}\right)}^{i}-x_{\left(n_{x}\right)}}{{{\alpha \sigma }_{\left(n_{x}\right)}}^{2}}\right)K_{\left(n_{x}\right)}\left(x_{s,\left(n_{x}\right)}^{i},x_{\left(n_{x}\right)}\right) \end{array}\right] \end{array}$$

We note that the most important difference between the scalar (Equation (16)) and the matrix‐valued (Equation (20)) kernel is that, in the scalar kernel, the value of kernel is the same for all components of the two given particles, while that is typically not true for the matrix‐valued kernel. The value of the scalar kernel can be seen as a generalized distance between particles. In other words, we only measure a single distance in the whole state space between two particles using the scalar kernel, while we independently measure the distances in each component using the matrix‐valued kernel. (That is, we obtain *n*_*x*_ distances between two particles in the matrix‐valued kernel.)

The differences in particle flow from using these two kernels become apparent when the convergence rate between components of the state is very different, which is the case when we only partially observe the system. Specifically, the convergence rate for the observed components is usually much faster than that for the unobserved components. In this case, even though the particles become very close in the observed component, the distance between two particles in terms of the full states can still be large because of the unobserved components, and hence, the value of the kernel can still be small when using the scalar kernel. In contrast, when the matrix‐valued kernel is used, each component in the state vector feels its closeness of the corresponding component in the other particle. Specifically, the observed components feel each other independent of the unobserved components, and the kernel value on the observed component can be large when the observed components are close.

In addition to the value of the kernel, the divergence of the kernel also makes the particle flows from two kernels different. Specifically, for the scalar kernel, the divergence of the kernel (Equation (19)) is proportional to the value of the kernel itself. Each component of the divergence of the kernel is scaled by the same kernel value. When the observed components of the two particles become very close, the value of the kernel can still be negligible because of the contribution of the unobserved components, as discussed in the last paragraph. This means that the magnitude of the divergence of the kernel is also negligible. Therefore, the repelling force is too weak to separate the particles away from each other in the observed component using the scalar kernel, leading to particles collapsing toward the mode in the observed component. On the other hand, for the matrix‐valued kernel, the value of each component in the divergence of the kernel (Equation (23)) is only proportional to how distance between two particles on this component behaves, and hence, the repelling force on the observed components can be effective.

**FIGURE 2:**
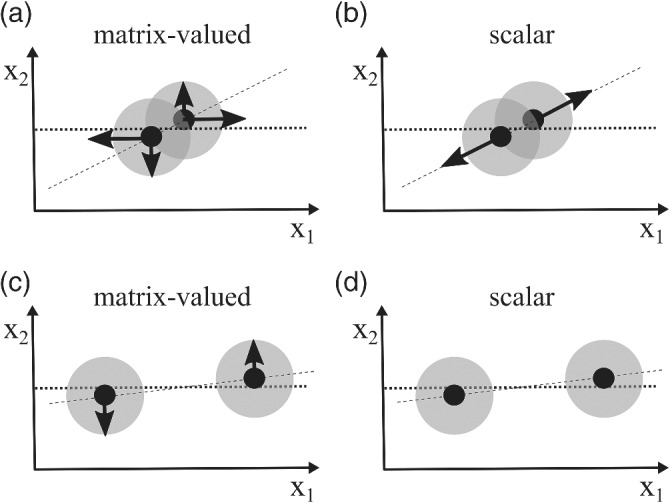
Comparison of the divergence of kernel between (a, c) the matrix‐valued kernel and (b, d) the scalar kernel. The shading is the scalar kernel (which is assumed to be an independent Gaussian with equal variance), the black dots are the particles, and the black arrows are the repelling forces resulting from the divergence of kernel. x1 denotes an unobserved component, and x2 is an observed component. (a, b) Divergence for both kernels when the convergence rate of observed and unobserved components is similar, leading to similar repelling forces. (c, d) Divergence for both kernels when the convergence rate of the observed component is larger than that of the unobserved component. For the scalar kernel, the repelling force is small in all directions, such that the particles collapse to the posterior mode in the observed component. The matrix‐valued kernel shows the correct behavior with a strong repellent force for the observed component because the divergence is allowed to be very different for the different components

Figure 2 shows a two‐dimensional example of the difference in behavior of the two kernels. Suppose x_1_ is the unobserved component and x_2_ is the observed component. When x_1_ and x_2_ converge at a similar rate so that two particles become close in both directions at the same time, the difference of the repelling force (i.e., the divergence of kernel) between the two kernels is small (Figure 2a,b). This can occur when the observation has a strong impact on (or is highly correlated with) the update of the unobserved variable. When the unobserved component converges much slower than the observed component in Figure 2c,d, the repelling force of the scalar kernel remains small even though the observed components are very close (Figure 2d), leading to collapse of the particles onto the posterior mode in that direction. However, the matrix‐valued kernel with independent repelling force in each component leads to a strong repelling force on the observed components (Figure 2c), avoiding the collapse on the posterior mode. We note that the divergence of the kernel is not only dependent on the kernel value but also on the factor in front, but the value of the kernel part turns out to be most important.

**FIGURE 3:**
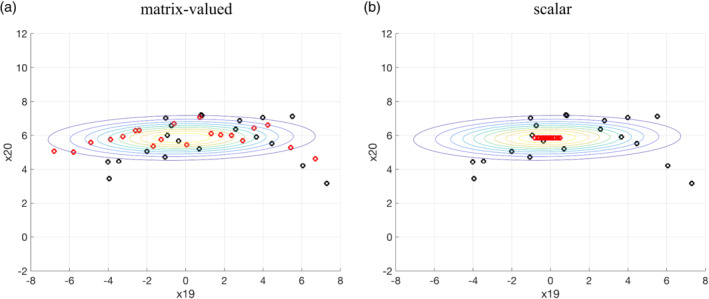
The prior and posterior marginal distribution of the variable x_(19)_ (unobserved component) and x_(20)_ (observed component) before and after the first data assimilation update (*t* = 20) with linear observation operator defined in Equation (30), using the (a) matrix‐valued kernel (b) scalar kernel. The black circles are the particles for the prior, red circles are the particles for the posterior, and the contour is the posterior covariance given by the ensemble Kalman filter [Colour figure can be viewed at wileyonlinelibrary.com]

We demonstrate the difference of the posterior solution using the scalar and matrix‐valued kernel in the 1,000‐dimensional Lorenz 96 system detailed in Section 3. Figure 3 shows the posterior marginal distribution of the variable x_(19)_ (unobserved component) and x_(20)_ (observed component) after the first data assimilation update. This is similar to the case in Figure 2c,d: the matrix‐valued kernel is able to keep particles away from each other, preventing the collapse in the observed component (Figure 3a), while the particles collapse for x_(20)_ using the scalar kernel (Figure 3b).

As a final note, the matrix‐valued kernel can be more general than the diagonal version we explore here. Off‐diagonal elements could be used to communicate the repelling force from one grid point to its neighbors. We did not need that in the experiments below, but it is an interesting path of research for the future.

### 2.4 Implementation of the PFF

Algorithm 1 summarizes the steps for implementing the PFF. We can use the gradient descent method to push the particles so that each particle is moved along the direction of the steepest descent of the KL divergence. However, the convergence rate using the gradient descent is too slow. We use a quasi‐Newton method with the prior covariance as preconditioner to speed up convergence (see, e.g., Nocedal and Wright, 2006). The calculation of the prior covariance is required in the PFF, so there is no additional cost if we choose the prior covariance as the preconditioner.

Another advantage of using the prior covariance as the preconditioner is that it helps to maintain the dynamical balance between the state variables. It is important in the numerical weather prediction model that the initial condition after the data assimilation update should stay in a physically realistic regime and “balanced,” meaning that the update does not put the system in an unbalanced state resulting in a rapid transition immediately after the update, for example, by producing unrealistic gravity waves, which will affect the quality of the forecast.

Algorithm 1 The algorithm below is provided to aid implementation for low‐to‐moderate dimensional systems. For high‐dimensional systems, modifications will be needed to make the implementation more efficient

[Input to the algorithm]

$x_{0}^{i}\;\left(i=1,\ldots ,N_{p}\right)$ [the prior ensemble] (*N*_*x*_ × 1)

**y** [observation] (*N*_*y*_ × 1)

**R** [observation error covariance] (*N*_*y*_ × *N*_*y*_)

[Assume a Gaussian prior in this pseudo code]

${\bar{x}}_{0}\leftarrow \frac{1}{N_{p}}\sum_{i=1}^{N_{p}}x_{0}^{i}$ [ensemble mean] (*N*_*x*_ × 1)

$X\leftarrow \left[x_{0}^{1}-{\bar{x}}_{0},x_{0}^{2}-{\bar{x}}_{0},\ldots ,x_{0}^{N_{p}}-{\bar{x}}_{0}\right]$ [ensemble perturbation matrix] (*N*_*x*_ × *N*_*p*_)

$B\leftarrow \frac{1}{N_{p}-1}XX^{T}$ [prior covariance matrix] (*N*_*x*_ × *N*_*x*_)

**B ← B** ∘ **C** [localization, see Equations (28) and (29)] (*N*_*x*_ × *N*_*x*_)

s = 0 [pseudo time for the data assimilation]

repeat

for i = 1,…,*N*_*p*._

$y^{i}\leftarrow H\left(x_{s}^{i}\right)$ [modeled observation] (*N*_*y*_ × 1)

$H^{i}\leftarrow \frac{\mathrm{dH}}{dx}\left(x_{s}^{i}\right)$ [linearized observation operator] (*N*_*y*_ × *N*_*x*_)

$\nabla \text{logp}\left(x_{s}^{i}|y\right)\leftarrow {H^{i}}^{T}R^{-1}\left(y-y^{i}\right)-B^{-1}\left(x_{s}^{i}-{\bar{x}}_{0}\right)$ [gradient of log posterior] (*N*_*x*_ × 1)

end for

for *d* = 1,…,*N*_*x*_

for *i* = 1,…,*N*_*p*_

${f_{s}}_{\left(d\right)}^{i}=0$ [the (*d*)‐th component of the particle flow for the *i*‐th particle] (1 × 1)

$x_{\left(d\right)}^{i}\leftarrow {e_{\left(d\right)}}^{T}x_{s}^{i}$ [the (*d*)‐th component of $x_{s}^{i}$] (1 × 1)

${\frac{\partial p}{\partial x}}_{\left(d\right)}^{i}\leftarrow {e_{\left(d\right)}}^{T}\nabla \text{logp}\left(x_{s}^{i}|y\right)$ [the (*d*)‐th component of $\nabla \text{logp}\left(x_{s}^{i}|y\right)$] (1 × 1)

for j = 1,…,*N*_*p*_

$x_{\left(d\right)}^{j}\leftarrow {e_{\left(d\right)}}^{T}x_{s}^{j}$ [the (*d*)‐th component of $x_{s}^{j}$] (1 × 1)

$K_{\left(d\right)}^{i,j}\leftarrow \exp\left(-\frac{1}{2}\frac{{\left(x_{\left(d\right)}^{i}-x_{\left(d\right)}^{j}\right)}^{2}}{\alpha B_{d,d}}\right)$ [the kernel Equation (21)] (1 × 1)

${\frac{\partial K}{\partial x}}_{\left(d\right)}^{i,j}\leftarrow -\left(\frac{x_{\left(d\right)}^{i}-x_{\left(d\right)}^{j}}{\alpha B_{d,d}}\right)K_{\left(d\right)}^{i,j}$ [the gradient of kernel Equation (23)] (1 × 1)

${I_{f}}_{\left(d\right)}^{i}\leftarrow {I_{f}}_{\left(d\right)}^{i}+\frac{1}{N_{p}}\left(K_{\left(d\right)}^{i,j}{\frac{\partial p}{\partial x}}_{\left(d\right)}^{i}+{\frac{\partial K}{\partial x}}_{\left(d\right)}^{i,j}\right)$ [integral part of Equation (6)] (1 × 1)

end for

end for

end for

for i = 1,…,*N*_*p*_

$f_{s}^{i}\leftarrow BI_{f}^{i}$ [multiply integral by **B** to find particle flow Equation (6)] (*N*_*x*_ × 1)

$x_{s}^{i}\leftarrow x_{s}^{i}+\text{∆}sf_{s}^{i}$ [Equation (7)] (*N*_*x*_ × 1)

end for

*s* ← *s* + 1

until stopping criterion met

[output of the algorithm]

$x_{s}^{i}\;\left(i=1,\ldots N_{p}\right)$ [the posterior ensemble members] (*N*_*x*_ × 1)

The pseudo time step (the time step for the iterations in Equation (7)) ∆*s* should be small enough to prevent instability of the iterative procedure. We use an adaptive scheme to determine the pseudo time step ∆*s* to accelerate the convergence. In general, we start with a small ∆*s* at the beginning, and gradually increase ∆*s* during the iterations. This is because at the beginning of the iterations the magnitude of the particle flow is large and we need a smaller ∆*s* to ensure that the trajectories of the particles do not cross in state space. At later iterations, the magnitude of the particle flow becomes smaller, so we can use a larger ∆*s* to accelerate the convergence. Specifically, we start with a small initial ∆*s* based on trial and error, ensuring that the particle flow will not blow up in the first few iterations. Then, if the particle flow decreases for 20 pseudo time steps, we increase ∆*s* by a factor of 1.4. If the magnitude of the particle flow increases, we will decrease ∆*s* by a factor of 1.4. The initial ∆*s* is chosen differently for different observation types. For the linear and absolute value observation, the initial ∆*s* is 0.05, and for the square and exponential observation, the initial ∆*s* is 0.001. More research is needed on how to accelerate the convergence in particle flows, but the practical scheme outlined above works well for our problem.

## 3 EXPERIMENTAL DESIGN

We compare the effect of the PFF and the local ensemble transform Kalman filter (LETKF) by applying both methods to the Lorenz 96 model. The equation for the *n*_*x*_‐dimensional Lorenz 96 model is

(24) $$\frac{dx_{\left(a\right)}}{\mathrm{dt}}=\left(x_{\left(a+1\right)}-x_{\left(a-2\right)}\right)x_{\left(a-1\right)}-x_{\left(a\right)}+F$$

where a = 1, …, *n*_*x*_ and *x*_(*a*)_ is the *a*‐th component of the state **x** defined in Equation (22). We set *n*_*x*_ to 1,000 and F to 8. The fourth‐order Runge–Kutta scheme is used, and the time resolution is ∆t = 0.01. The initial condition is set as

(25) $$x_{\left(a\right)}\left(t=0\right)=\left\{\begin{array}{c} F,\;\text{if}\;\mathrm{mod}\left(a,5\right)\;\neq 0\\ F+1\;\text{if}\;\mathrm{mod}\left(a,5\right)\;=0 \end{array}\right.$$

and the model is integrated for 1,000 time steps to generate chaotic behavior. At t = 1,000, *N*_*p*_ = 20 ensemble members are generated by adding random perturbations, following a normal distribution $N\left(0,2I_{n_{x}\times n_{x}}\right)$. A run without the random perturbation at t = 1,000 is taken as the truth. After then, observations with random noise, following *N*(**0**, **R**), where $R=\epsilon I_{n_{y}\times n_{y}}$ (*ϵ* will be different for different observation types) and *n*_*y*_ is the number of observations, are assimilated into the system every 20 time steps (which roughly corresponds to every 24 hr in atmospheric models). The magnitude of the observational error *ϵ* depends on the observation operators. The observation is taken at every fourth variable in the system, which means only 25% of the system is observed and $n_{y}=\frac{n_{x}}{4}$. Note that this is a fixed‐observing system, meaning that 75% of the system is never observed. The ensemble and the truth are integrated for 1,500 time steps.

To obtain a more statistically reliable comparison between the PFF and the LETKF, the experiments described above are averaged over 10 different random realizations of the ensemble perturbations, the truth, and the observation errors for both the PFF and the LETKF. The only exception is for the square observation, in which case we are not able to find suitable parameters for the LETKF for 9 of the 10 realizations. In other words, the model blows up before 1,500 time steps for these nine realizations when using LETKF to assimilate the square observation. Nevertheless, the model remains stable using the PFF to assimilate square observations for all the 10 realizations. Therefore, when comparing the performance of the LETKF and the PFF for assimilating the square observation, the performance for the LETKF is evaluated by only one realization, while for the PFF is by all the 10 realizations. For other observations, we will compare the averaged performance of the results from all the 10 realizations in the following.

For both the PFF and the LETKF, we need to localize the effect of the observations due to the fact that the dimension of system is much larger than the sample size. For the LETKF, we update the system grid point by grid point, using the localized observational error covariance **R**_*i*_ when updating the *i*‐th grid point:

(26) $$R_{i}=R\circ C_{i}$$

where ∘ is the Schur product of the observational error covariance and the matrix **C**_*i*_:

(27) $$\begin{array}{c} C_{i}=\mathrm{diag}\left(\left[\exp\left\{-{\left(\frac{d\left(i,1\right)}{r_{\text{in}}}\right)}^{2}\right\},\right.\right.\\ \exp\left\{-{\left(\frac{d\left(i,2\right)}{r_{\text{in}}}\right)}^{2}\right\},\ldots ,\\ \left.\left.\exp\left\{-{\left(\frac{d\left(i,n_{y}\right)}{r_{\text{in}}}\right)}^{2}\right\}\right]\right) \end{array}$$

where *d*(*i*, *j*) is the distance between the *i*‐th grid point and the *j*‐th observation, and *r*_*in*_ is the decorrelation length scale for the observation, which is set to *r*_*in*_ = 4. The choice of the decorrelation length scale *r*_*in*_ is based on the properties of the Lorenz 96 system. We retain the covariance between the state variables that are within three times the decorrelation length scale from the observation location. That is, 25 (= 1 + 2 × 3 × *r*_*in*_) state variables survive this localization.

For the PFF, we assume a Gaussian prior in the standard experiment described here. We directly localize on the prior covariance matrix:

(28) B←B∘C

where

(29) $$C={\left[c_{i,j}\right]}_{n_{x}\times n_{x}},c_{i,j}=\exp\left\{-{\left(\frac{i-j}{r_{\text{in}}}\right)}^{2}\right\}$$

Note that *r*_*in*_ is also set as 4 here.

We compare the performance of the PFF and the LETKF using different types of observations, including linear and nonlinear observations. The linear observation operator is

(30) $$H_{\text{linear}}\left(x\right)={\left[\begin{array}{c} \begin{array}{c} x_{\left(4\right)}\\ x_{\left(8\right)}\\ \vdots \end{array}\\ x_{\left(n_{x}\right)} \end{array}\right]}_{n_{y}\times 1}$$

The observational error is set as *ϵ* = 0.5. For the nonlinear observations, we consider several observational operators: absolute value, exponential, and square operator. For the absolute value operator, which is

(31) $$H_{\mathrm{abs}}\left(x\right)={\left[\begin{array}{c} \begin{array}{c} \left|x_{\left(4\right)}\right|\\ \left|x_{\left(8\right)}\right|\\ \vdots \end{array}\\ \left|x_{\left(n_{x}\right)}\right| \end{array}\right]}_{n_{y}\times 1}$$

the pdf of the likelihood as function of the state will be bimodal. The magnitude of the linearized observational operator will be independent of the state. The observational error is set as *ϵ* = 0.5. We will also test the behavior of the methods for an exponential observation operator, given by

(32) $$H_{\exp}\left(x\right)={\left[\begin{array}{c} \begin{array}{c} e^{\frac{x_{\left(4\right)}}{6}}\\ e^{\frac{x_{\left(8\right)}}{6}}\\ \vdots \end{array}\\ e^{\frac{x_{\left(n_{x}\right)}}{6}} \end{array}\right]}_{n_{y}\times 1}$$

leading to a unimodal likelihood. However, the magnitude of the linearized observational operator will depend on the state. The observational error is set as *ϵ* = 0.01. Finally, for the squared operator, given by

(33) $$H_{\text{square}}\left(x\right)={\left[\begin{array}{c} \begin{array}{c} {x_{\left(4\right)}}^{2}\\ {x_{\left(8\right)}}^{2}\\ \vdots \end{array}\\ {x_{\left(n_{x}\right)}}^{2} \end{array}\right]}_{n_{y}\times 1}$$

the pdf of the likelihood as function of the state will be bimodal, and the magnitude of the linearized observation operator will depend on the state, which is the most complicated operator. The observational error is set as *ϵ* = 1.

## 4 RESULTS

The performance of the PFF is tested in a sequential data assimilation experiment as described in Section 3. To compare the results between the PFF and the LETKF, the prior for the PFF is assumed to be Gaussian, which is the same assumption as is used in the LETKF. The difference between the PFF and the LETKF is then in the likelihood. When the observation is linear, their performance is expected to be similar. However, when the observation is nonlinear, the non‐Gaussian likelihood is expected to cause differences in the two methods.

### 4.1 Linear observation operator

To quantitatively compare the results from different data assimilation (DA) methods, the root mean square error (RMSE) of all the variables (total RMSE) is used to evaluate their performance:

(34) $$\text{RMSE}\text{\_}X\left(t\right)=\sqrt{\frac{1}{n_{x}}\sum_{a=1}^{n_{x}}{\left({\bar{x}}_{\left(a\right)}\left(t\right)-{x_{t}}_{\left(a\right)}\left(t\right)\right)}^{2}}$$

where ${\bar{x}}_{\left(a\right)}$ is the ensemble mean for the *a*‐th component of the state vector $\bar{x}$ and *x*_*t*(*a*)_ is the *a*‐th component of the truth. We also compare the RMSE of the observed variables alone for different experiments, which is

(35) $$\text{RMSE}\text{\_}X\text{\_}\mathrm{OBS}\left(t\right)=\sqrt{\frac{1}{\left|\mathrm{OBS}\right|}\sum_{\left(a\right)\in \mathrm{OBS}}{\left({\bar{x}}_{\left(a\right)}\left(t\right)-{x_{t}}_{\left(a\right)}\left(t\right)\right)}^{2}}$$

where *OBS* = {4, 8, …, *n*_*x*_} is the set containing the indices of the observed variables and |*OBS*| is the number of the elements in the set *OBS* ($\left|\mathrm{OBS}\right|=n_{y}=\frac{n_{x}}{4}$). Similarly, the RMSE of the unobserved variables is

(36) $$\begin{array}{c} \text{RMSE}\text{\_}X\text{\_}\text{NOOBS}\left(t\right)\\ =\sqrt{\frac{1}{\left|\text{NOOBS}\right|}\sum_{\left(a\right)\in \text{NOOBS}}{\left({\bar{x}}_{\left(a\right)}\left(t\right)-{x_{t}}_{\left(a\right)}\left(t\right)\right)}^{2}} \end{array}$$

where *NOOBS* = {1, 2, …, *n*_*x*_} ∖ *OBS* and $\left|\text{NOOBS}\right|=n_{x}-n_{y}=\frac{3}{4}n_{x}$.

**FIGURE 4:**
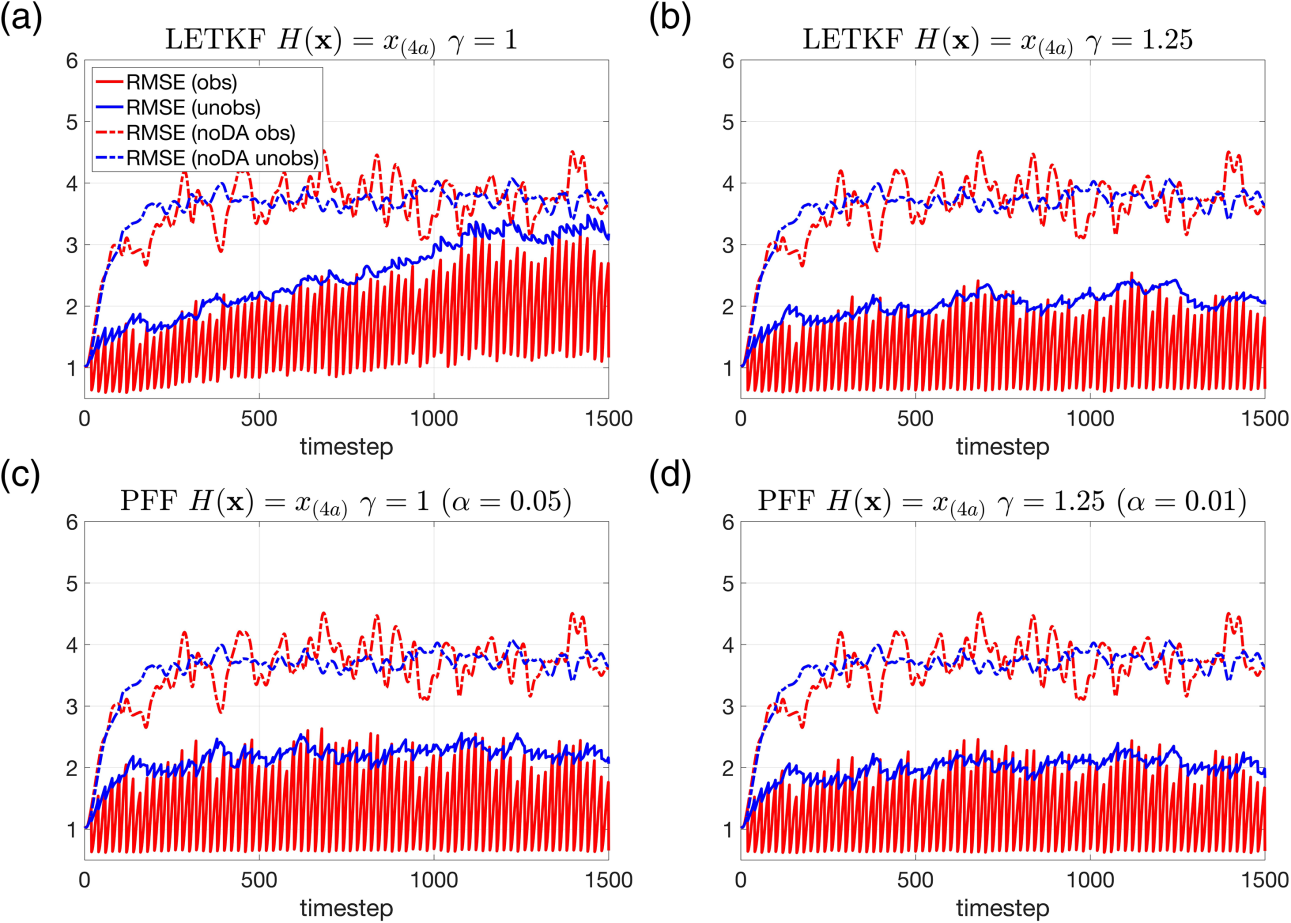
Root‐mean‐square error (RMSE) of the noDA ensemble (i.e., the ensemble with same initial conditions but without data assimilation, dashed lines) and with data assimilation (solid lines) using (a) the LETKF without inflation γ = 1, (b) the LETKF with inflation factor γ = 1.25, (c) the PFF without inflation γ = 1 and with kernel width α = 0.05, and (d) the PFF with inflation factor γ = 1.25 and with kernel width α = 0.01 [Colour figure can be viewed at wileyonlinelibrary.com]

Figure 4 shows the RMSE of the LETKF and the PFF results. Both methods show a reduced RMSE for both observed and unobserved variables compared with the ensemble without data assimilation (noDA ensemble). For the LETKF without inflation, Figure 4a shows that its RMSE of the observed variables decreases at the observation times. The RMSE of the unobserved variables also decreases at most of the observation times before *t* = 800, while it increases, instead, for most of the observation times after *t* = 800. This suggests that the covariance between observed and unobserved variables is not good enough to update the unobserved variables correctly after *t* = 800 for the LETKF without inflation. In addition, the RMSE of the observed variables at the observation times gradually increases with time. This suggests that the system is gradually biased against the observations.

After extensive experimentation, we found the best value for the inflation factor was 1.25 for the LETKF. Figure 4b shows that the RMSE of the observed variables decreases to around 0.6–0.7 at almost all the observation times. The RMSE of the unobserved variables also decreases for most of the observation times. This suggests that with the inflated prior, the covariance structure becomes better and LETKF is able to better follow the system without the possible filter divergence as in Figure 4a.

With a proper choice of the kernel width α in Equation (21), we are able to have a stable RMSE of the observed variables for the PFF either with or without inflation of the prior (Figure 4c,d). However, it is found that with the inflation of the prior in the PFF, the RMSE of both observed and unobserved variables still slightly decreases. Comparing the performance of the LETKF with inflated prior (Figure 4b) and the PFF with inflated prior (Figure 4d), we find their RMSE of the observed variables comparable, while it is interesting to note that the PFF has a slightly smaller RMSE of the unobserved variables than the LETKF does. Generally, Figure 4 suggests that the performance of the PFF is overall comparable to a well‐tuned LETKF when the observation is linearly related to the model state.

In addition to the behavior of the mean, the “reliability” of the ensemble is also important. A “reliable ensemble” can defined as the ensemble in which “the truth and the forecast ensemble can be considered samples from the same probability distribution.” (Hamill, 2001). In other words, the distribution represented by the ensemble is indistinguishable from the distribution from which the truth is drawn. To evaluate the reliability, the rank histogram is used as another measure of performance. A rank histogram close to a uniform distribution is considered as a necessary condition for a reliable ensemble.

**FIGURE 5:**
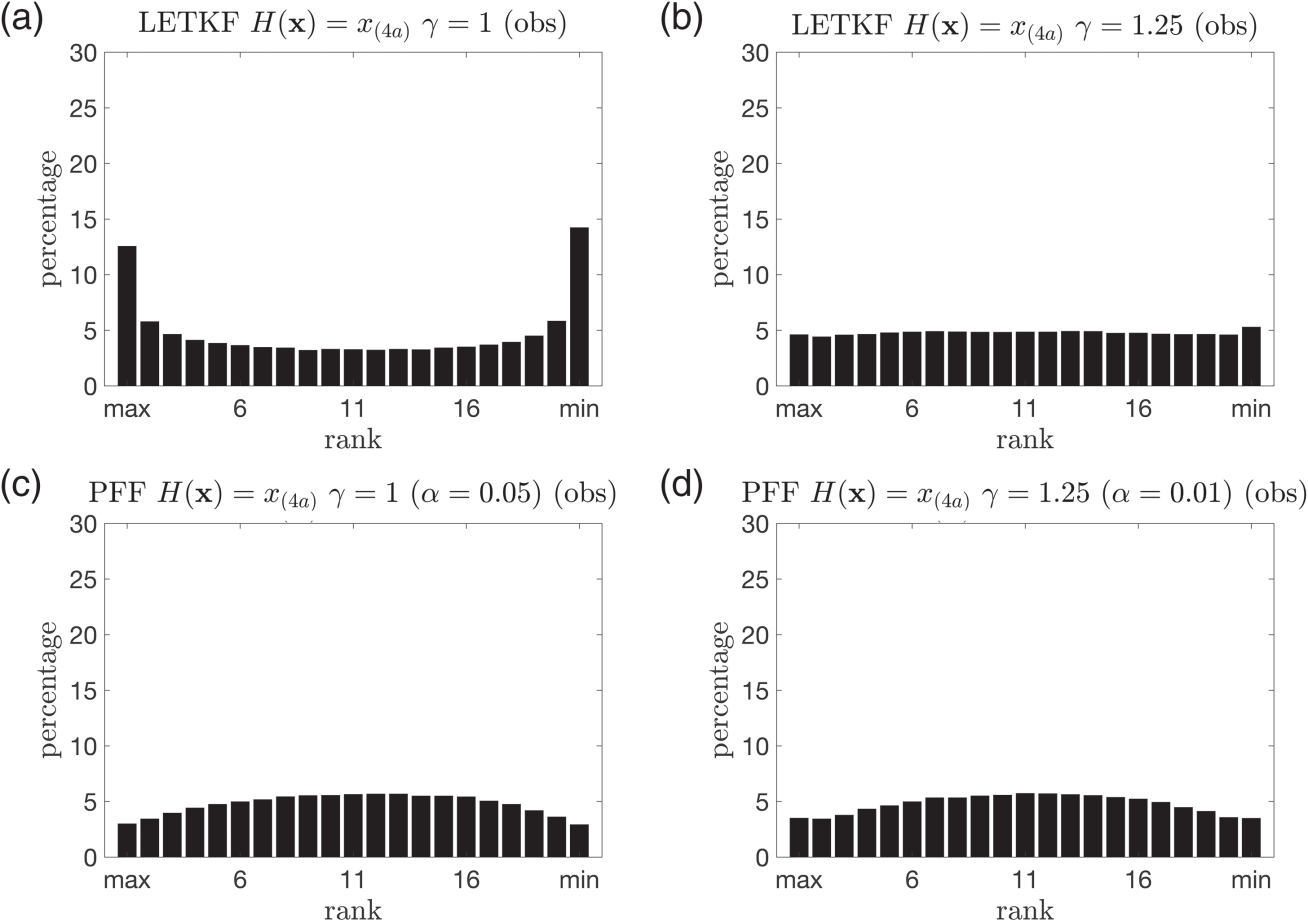
Rank histogram of the observed variables for the prior compared with the truth at the observation times for the linear observation. The ensemble from (a) the LETKF without inflation γ = 1, (b) the LETKF with inflation factor γ = 1.25, (c) the PFF without inflation γ = 1 and with kernel width α = 0.05, and (d) the PFF with inflation factor γ = 1.25 and with kernel width α = 0.01

The rank histogram of the observed variables in the prior compared with the truth at the observation times is shown in Figure 5. It is shown that the rank histogram for the LETKF without inflation (Figure 5a) is close to U‐shape, suggesting that the ensemble may be either biased against the truth or underdispersive. Based on Figure 4a, since the RMSE of the observed variables at observation times gradually increases with time, it can be inferred that the ensemble is also gradually biased against the truth. With inflation of the prior, the rank histogram from the LETKF becomes flat (Figure 5b). The rank histogram from the PFF is quite close to the uniform distribution, despite that the ensemble is slightly overdispersive (Figure 5c,d). The rank histogram for the unobserved variables is similar to that of the observed variables (not shown).

In a brief summary, we find that, for the linear observation, the PFF shows comparable results to the LETKF with prior inflation. In the setup used here, both the PFF and the LETKF assume a Gaussian prior, and the observation operator is linear. In this case, we would expect that PFF shows similar results with a well‐tuned LETKF. Note that both inflating the prior and tuning the kernel width (see Section 5.1) for the PFF can make the posterior wider, to prevent filter divergence. However, unlike the inflation for the prior, the tuning of kernel width will not change the position of the mean in the posterior. In other words, the tuning of the kernel width retains most of the information from both the prior and the observations. Note that when there is evidence that the prior is underdispersive, we can still inflate the prior for the PFF. This suggests that the PFF is more flexible than the LETKF.

### 4.2 Nonlinear observation operators

When the model states are nonlinearly related to the observations, the likelihood is no longer Gaussian as a function of the model state, making the posterior non‐Gaussian too. In this case, the mean of the ensemble model state may not be representative of the behavior of the ensemble, and so a RMSE in the model state may not be a useful measure of performance, for example, when the posterior is a multimodal distribution. This happens for the square observation *H*(**x**) = *x*_(4*a*)_^2^, where the observation operator is not one‐to‐one even if we confine the domain to the subset of the observed variable. To measure the performance of the ensemble for the observed variables, instead, we compare the RMSE defined in the observational space. Given that the measurement error (the observation error in the observational space) is taken to be Gaussian, the posterior in the observational space is expected to be closer to Gaussian, and at least less likely to be multimodal. The RMSE of the observed variables defined in the observational space is

(37) $$\text{RMSE}\text{\_}Y\left(t\right)=\sqrt{\frac{1}{\left|\mathrm{OBS}\right|}\sum_{\left(a\right)\in \mathrm{OBS}}{\left({\bar{y}}_{\left(a\right)}\left(t\right)-{y_{t}}_{\left(a\right)}\left(t\right)\right)}^{2}}$$

where ${\bar{y}}_{\left(a\right)}$ is the ensemble mean of the modeled observation given by the *a*‐th component of the model state, as defined in Equation (38):

(38) $${\bar{y}}_{\left(a\right)}=\frac{1}{N_{p}}\sum_{i=1}^{N_{p}}H\left(x_{\left(a\right)}^{i}\right)$$

and *y*_*t*(*a*)_ is the modeled observation given by the *a*‐th component of the truth,

(39) $${y_{t}}_{\left(a\right)}=H\left(x_{t,\left(a\right)}\right)$$

**FIGURE 6:**
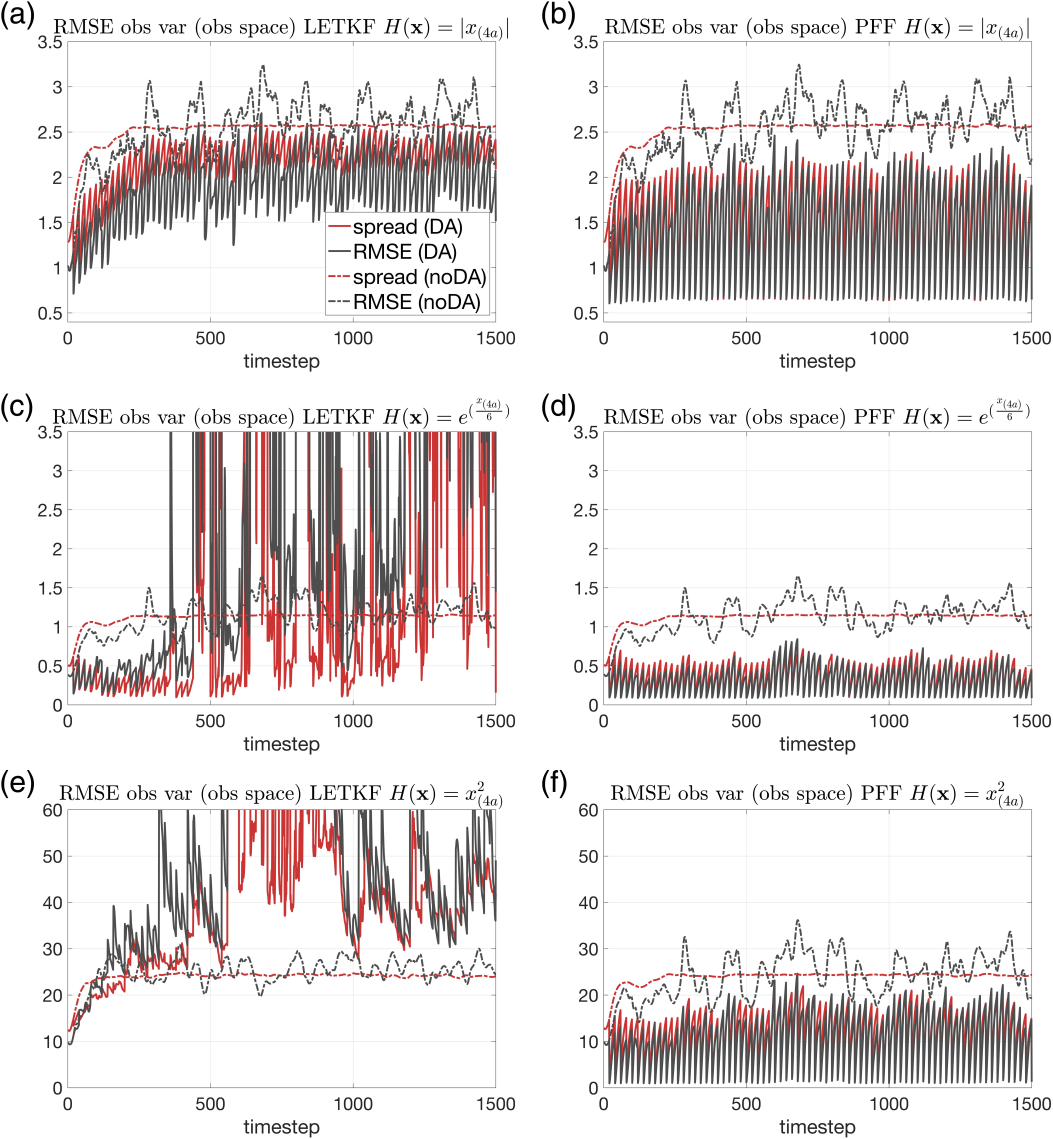
RMSE and the ensemble spread of the observed variables defined in the observational space for the nonlinear observations. The dashed lines are from the noDA ensemble, and the solid lines are from the (a, c, e) LETKF and (b, d, f) PFF. The observation operator is (a, b) the absolute value operator, (c, d) the exponential operator, and (e, f) the square operator [Colour figure can be viewed at wileyonlinelibrary.com]

Figure 6 shows the RMSE and the spread of the observed variables in the observational space for the nonlinear observations. For the absolute value operator, both the RMSE and the spread at observation times gradually increase with time and become steady after *t* = 300 for the LETKF, and the LETKF can still slightly improve the ensemble compared with the noDA ensemble (Figure 6a). For the exponential operator, the results from LETKF are not very stable: the results are good before *t* = 400, but after *t* = 400, the error sometimes grows very fast between the observation times and sometimes is even larger than the noDA ensemble (Figure 6c). Similar results can be found for the square operator for the LETKF (Figure 6e). In contrast, the performance of the PFF for these three nonlinear observations are all very good and stable (Figure 6b,d,f). All of the three experiments show an improvement over the noDA ensemble. We note that the parameters (inflation factor and the localization radius) in LETKF for these experiments are already the best: if the parameters are changed even a small amount, the model blows up before *t* = 1,500.

**FIGURE 7:**
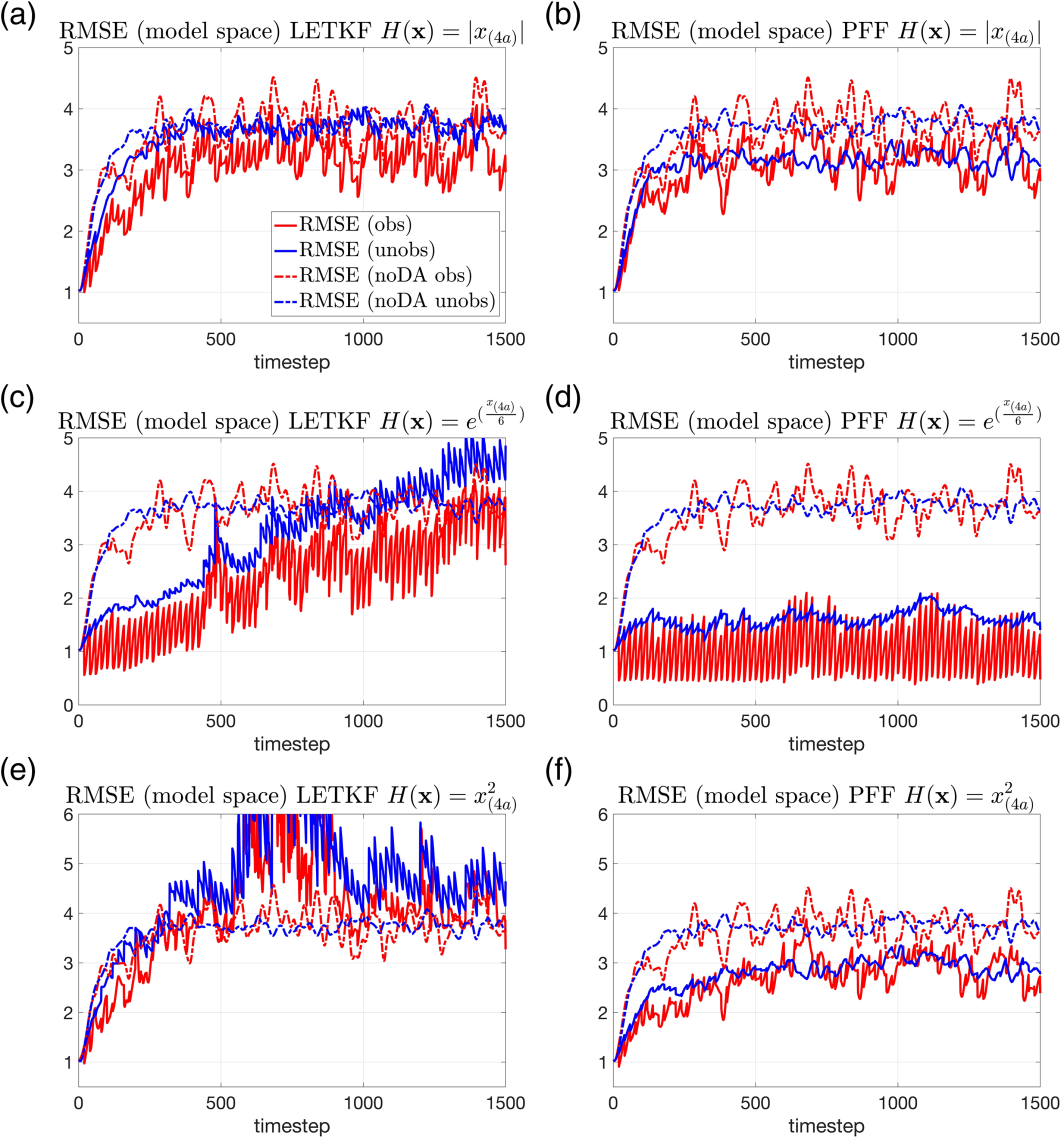
The same as Figure 3, but for the nonlinear observations. The solid lines are from the (a, c, e) LETKF and (b, d, f) PFF. The observation operator is (a, b) the absolute value operator, (c, d) the exponential operator, and (e, f) the square operator [Colour figure can be viewed at wileyonlinelibrary.com]

For the behavior of the unobserved variables, the LETKF only shows an improvement in the RMSE for the absolute value observation before *t* = 300 (Figure 7a), for the exponential observation before *t* = 1,000 (Figure 7c). After that, the RMSE from the LETKF is almost indistinguishable from the noDA ensemble for the absolute value operator (Figure 7a), and becomes even worse for the other two observational operators (Figure 7c,e). In contrast, the RMSE from the PFF shows an improvement for all the observational operators over the noDA ensemble (Figure 7b,d,f). It is worth noting that for the PFF, the RMSE of the observed variables in model space for the absolute value (Figure 7b) and square observations (Figure 7f) at observation times are quite variable, while their values are very stable when evaluated in the observation space (Figure 6b,f). This demonstrates the problem of using the ensemble mean as a measure of the performance when the posterior is possibly not unimodal in the model space.

**FIGURE 8:**
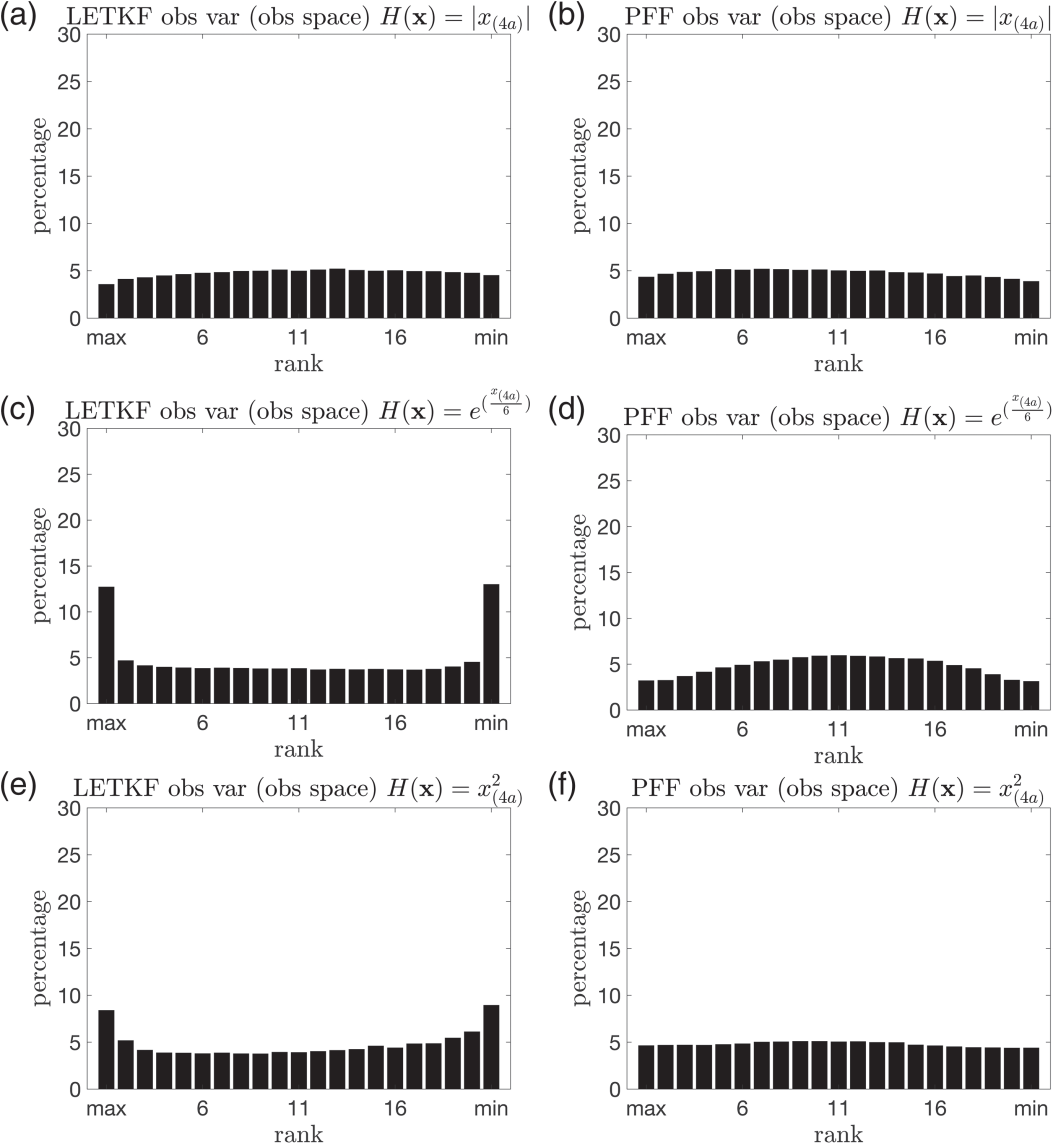
The same as Figure 5, but for the observed variables in the observational space for the nonlinear observations. The ensemble is from the (a, c, e) LETKF and (b, d, f) PFF. The observation operator is (a, b) the absolute value operator, (c, d) the exponential operator, and (e, f) the square operator

The rank histogram in the observational space is also used to verify the reliability of the ensemble for the nonlinear observations. Figure 8a,b shows that, for the absolute value operator, both the LETKF and the PFF have a rank histogram close to the uniform distribution. Note that the LETKF is not able to generate a bimodal posterior pdf; it can only “choose” one mode given the observation. The magnitude of the linearized observational operator is independent of the model state for the absolute value operator, meaning that this observation operator is equivalent to the linear operator when the model state is far from 0. Therefore, the incapability of generating the two modes of the posterior is a major error source for the LETKF when the observation is the absolute value operator. However, this error becomes less apparent when we transform the variables from the model state to the observational space. This can partially explain why the rank histogram of the LETKF in the observational space is close to the uniform distribution for the absolute value operator. However, for the other nonlinear observations, the rank histograms from the LETKF become U‐shaped (Figure 8c,e). This is a result of biased mean, as is evident in Figure 7c,e. This suggests that the dependence of the linearized observational operator on the state is a major reason for the biased mean in the observational space for the LETKF. For the PFF, Figure 8b,d,f shows that the rank histograms for all the nonlinear operators are close to the uniform distribution, although slightly overdispersive for the exponential operator.

**FIGURE 9:**
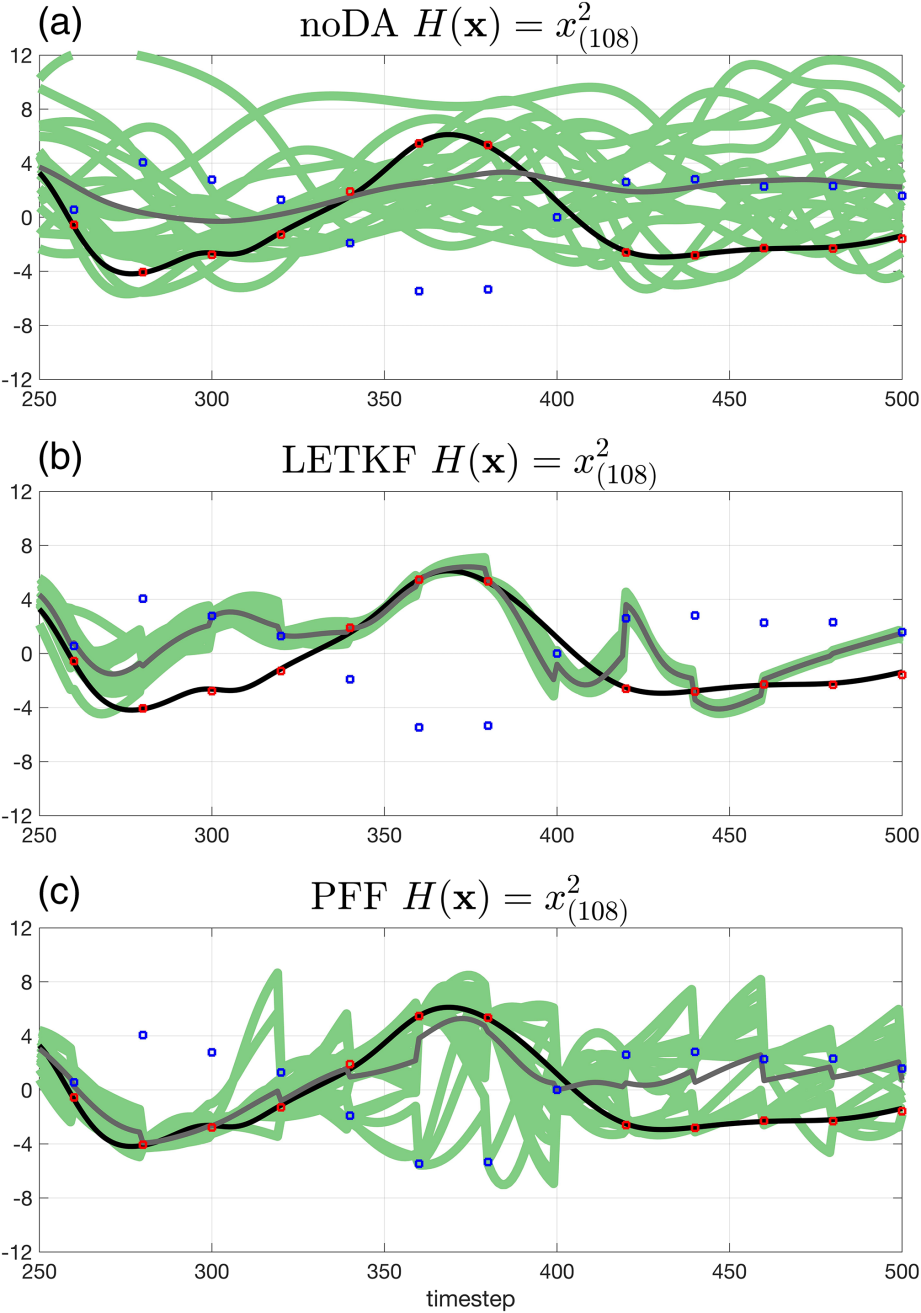
The time series of the observed variable *x*_(108)_ during *t* = 250–500. The black line is the truth, the green lines are the ensemble members, and the gray line is the ensemble mean. The square observation is assimilated into the system. The red dots are the solution with the same sign of the truth corresponding to the observations, and the blue dots are the second solution with the opposite sign of the truth corresponding to the observations. The ensemble is from (a) noDA, (b) LETKF, and (c) PFF [Colour figure can be viewed at wileyonlinelibrary.com]

To demonstrate the ability of PFF to generate multimodal posterior, we compare the time series of one of the observed variables *x*_(108)_ with square observations during *t* = 250–500 in Figure 9. Note that there can be two solutions, with the same magnitude but opposite in sign in the state space, given a square observation. We will call the solution with the opposite sign of the truth the second solution (blue dots in Figure 9). We note that, in a bimodal situation like this, the correct posterior solution is an ensemble that covers both the true and the second solutions, while individual particles follow one or the other. Both methods demonstrate an improvement of the variable evolution compared with the noDA ensemble (Figure 9). For LETKF, the whole ensemble sometimes follows the correct solutions (red dots), for example, at *t* = 340, 360, 380, 440, and 460, while the whole ensemble follows the second solution (blue dots) as well, for example, at *t* = 300, 320, 420, and 500 (Figure 9b). In contrast, for the PFF, the ensemble is able to follow both the true and the second solution at the same time (Figure 9c).

**FIGURE 10:**
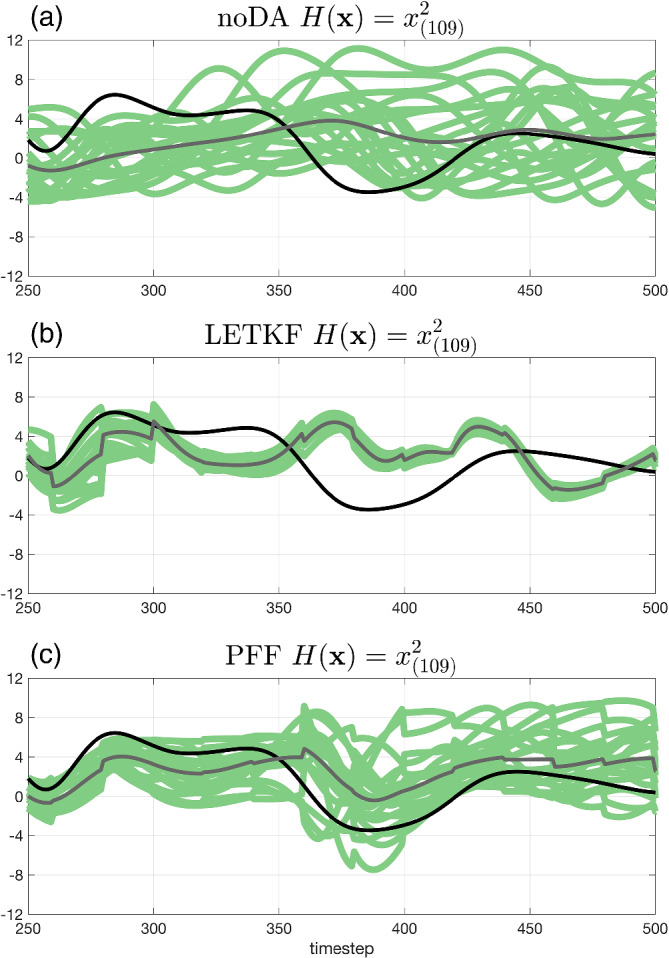
The same as Figure 9 but for the unobserved variable *x*_(109)_. The ensemble is from (a) noDA, (b) LETKF, and (c) PFF [Colour figure can be viewed at wileyonlinelibrary.com]

Note that when the whole ensemble from the LETKF chooses the wrong mode (i.e., follows the second solution) for the observed variables, the behavior of the unobserved variables will also be impacted by the poor covariance structures. Figure 10 shows the time series of the unobserved variable *x*_(109)_, whose update during the data assimilation is immediately affected by its neighbor *x*_(108)_. It is shown that after *t* = 300, the ensemble from the LETKF gradually loses track of the truth, and the spread becomes smaller (Figure 10b). Compared with the LETKF, the ensemble from the PFF has a larger spread, but follows the truth much better (Figure 10c). Although the overall spread of the unobserved variable for the PFF is large, it does not mean that the PFF loses the skill during the data assimilation. The evolution of each ensemble member still follows the truth better and in a more consistent way than the noDA ensemble (this can also be inferred from Figure 7f), suggesting that the ability of the PFF to capture the multimodal distribution of the posterior can also improve the update of the unobserved variables in its neighborhood.

It is worthwhile to mention that, in the square observation case (and other multimodal likelihood or posterior), the ensemble mean in the model state space may not be representative. For example, at *t* = 420 for the PFF (Figure 9c), almost half of the ensemble for the observed variable chooses the true solution, while another half chooses the second solution, leading to the ensemble mean being close to the average of the two solutions, where the true posterior pdf will have little probability mass. In contrast, the behavior of the observed variable in each ensemble member from the LETKF is very similar (Figure 9b). In this case, the ensemble mean in the model state space is considered to be representative. To summarize, given the capability of PFF to generate the ensemble with multimodal distribution, we should be more cautious when using the ensemble mean in the model state space as a tool to diagnose or analyze the behavior of the ensemble. As a final note, while the posterior pdf is bimodal in observed variables, it has many more modes when considering all components together. In this sense, the behavior of the PFF is quite remarkable.

## 5 THE SENSITIVITY EXPERIMENTS

In this section, we conduct several preliminary sensitivity experiments examining the effect of different settings in the PFF, including the kernel width, the number of iterations, and the prior assumption on the performance of the PFF. The preliminary aspect is related to the fact that not much research has been conducted in this area, and the research areas are too vast to cover in this paper. The same experiment setups for the sequential data assimilation experiments as described in Section 3 are used.

### 5.1 The kernel width

We choose the kernel width α as the reciprocal of the number of the particles. The reason is twofold. First, we do not want α to be too large, as a large α means a strong smoothing of the particle flow through the weighting of the gradients with the kernel, in which case we lose the ability to describe the fine‐scale structures in the posterior pdf. On the other hand, we do not want α to be too small either, especially with only a limited number of particles. This is because a small α means the repelling force will be significant only when particles become very close. When we only have a limited number of particles, the particles are sparse in the space. Therefore, using a small α with a limited number of particles may cause the particles to almost collapse to the mode (i.e., become too close to the mode). Due to the above reasons, when we have more particles, we would like to make α smaller, but not too small, to keep the fine‐scale structures of the particle flow at the location where each particle lies. In other words, we would expect α to be inversely proportional to the number of particles.

**FIGURE 11:**
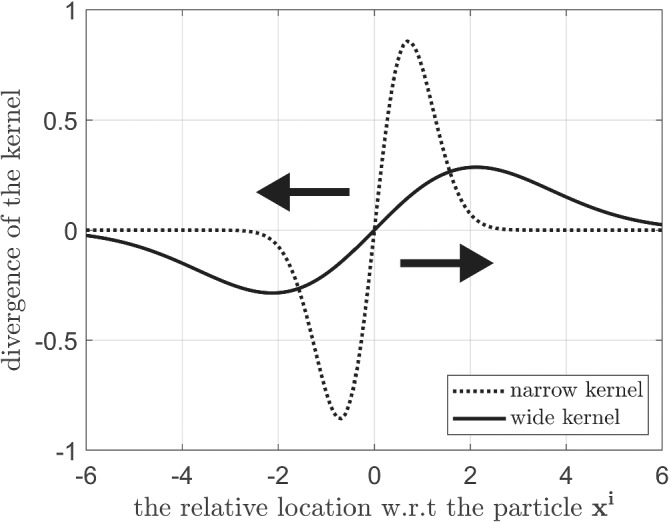
Demonstration of the effect of kernel width on the divergence of the kernel (repelling force). The system is assumed to be one‐dimensional. The *x* axis is the relative location with respect to one of the particles, and the *y* axis is the divergence of the kernel that is applied on the state whose particle flow is evaluated. The dashed (solid) line represents a small (large) kernel width. The arrow represents the direction of the repelling force

Indeed, the kernel width α is a tuning parameter, and there is no certain reason for why we choose it to be exactly the reciprocal of the number of particles. Therefore, the sensitivity of the performance of the PFF to α is examined in the following. Figure 11 demonstrates the effect of kernel width on the divergence of the kernel (i.e., the repelling force) for the Gaussian kernel we used in this study. Take a wider kernel (solid line in Figure 11), for example, although the range of influence for a particle is larger, the repelling force is generally smaller. This suggests that the particles can feel the repelling force from each other when their distance is large, which might lead to a larger spread of the posterior. However, the magnitude of the divergence of a wider kernel can also be too small to balance the gradient of logarithm of posterior, leading to a narrower posterior. In addition, we note that a larger kernel width means a stronger smoothing of the particle flow, which complicates the interpretation of the effect of the kernel width on the PFF.

**FIGURE 12:**
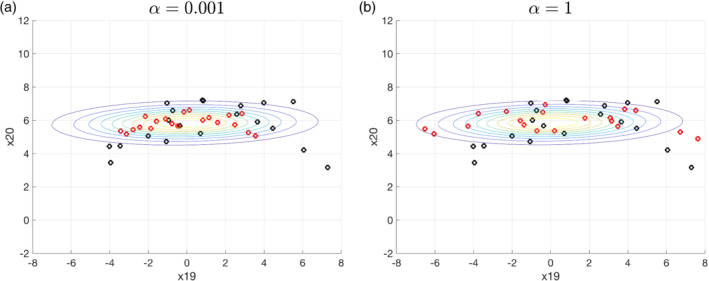
The same as Figure 3, but the kernels used here are both matrix‐valued kernels yet with different kernel widths. (a) A narrow kernel width α = 0.001. (b) A wide kernel width α = 1 [Colour figure can be viewed at wileyonlinelibrary.com]

**FIGURE 13:**
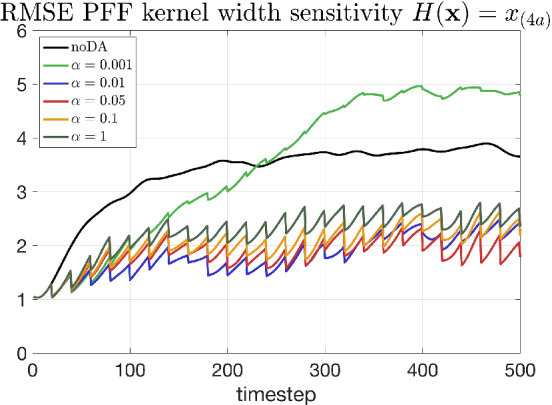
The total RMSE of the ensemble from the PFF, assimilating linear observations, with different kernel widths [Colour figure can be viewed at wileyonlinelibrary.com]

**FIGURE 14:**
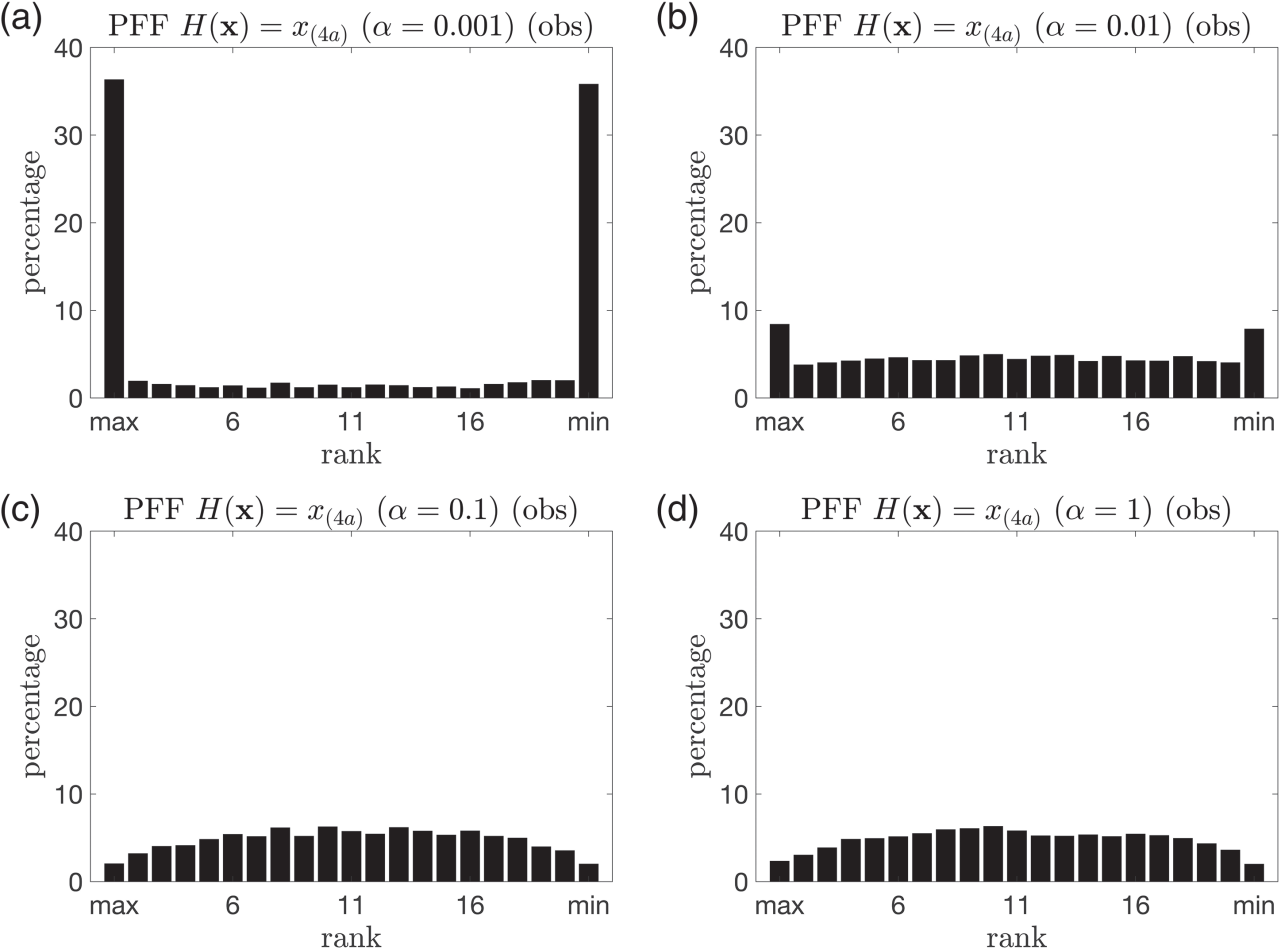
The same as Figure 5, but for the PFF with different kernel width. (a) α = 0.001, (b) α = 0.01, (c) α = 0.1, and (d) α = 1. For the experiment with α = 0.05, see Figure 5c

A set of sensitivity experiments are conducted to see what the effect of the kernel width is on the PFF, using linear observations and with all other settings the same as in Section 4 except for the kernel width. Figure 12 shows the posterior marginal distribution of the variable x_(19)_ (unobserved component) and x_(20)_ (observed component) after the first data assimilation update for a kernel with large kernel width and another with small kernel width. A larger kernel width results in a larger spread of the posterior, through the complicated interaction between particles (Figure 12b). Figure 13 shows the total RMSE of the ensemble for 500 time steps. It is found that, when the kernel width α is within the range [0.01, 0.1], the performance is comparable. When α is either too small or too large, the performance of PFF in terms of RMSE becomes suboptimal. In particular, when α = 0.001, the RMSE becomes larger than the noDA ensemble. Figure 14 shows the rank histogram of the prior for the observed variables at the observation times. For the smaller kernel width (Figure 14a,b), the rank histogram suggests the ensemble may be either biased against the truth or underdispersive. For larger kernel width (Figure 14c,d), the rank histogram suggests that the ensemble is slightly overdispersive. This suggests that a wider (narrower) kernel leads to a larger (smaller) spread of the posterior. The good news is that a range of a factor 10 for *α* still leads to good performance of the filter, so the PFF is not too sensitive to this parameter, at least for the experimental settings explored here.

### 5.2 The number of iterations

**FIGURE 15:**
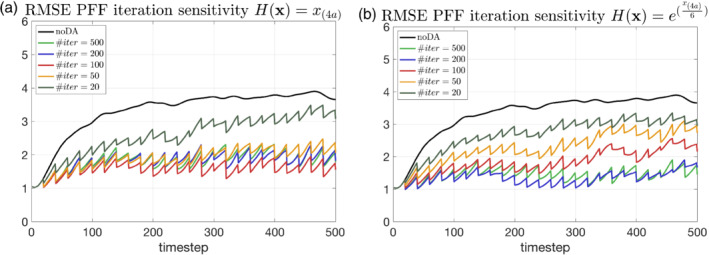
The same as Figure 13, but for a different number of iterations for the PFF using (a) the linear observation and (b) the exponential observation [Colour figure can be viewed at wileyonlinelibrary.com]

As in all iterative methods, we have to determine the number of iterations needed for successful interpretation of the PFF. The number of iterations is set to 500 for all previous experiments. We conduct two sets of sensitivity experiments, with one using linear observations and the other using exponential observations. Figure 15 illustrates this sensitivity by showing the total RMSE of the ensemble over 500 time steps. It is found that, once the number of iterations is over 50, the RMSE is quite similar for the linear observation case (Figure 15a). On the other hand, the RMSE becomes quasi‐steady only when the number of iterations is more than 200 for the exponential observation (Figure 15b). This result suggests that PFF requires more iterations for more complicated observation operators to converge to the steady‐state solution. Examinations of the iterations for PFF show that the number of iterations required can be different at different observation times (not shown). In addition, the error from the insufficient convergence at an earlier time can accumulate and affect the prior covariance at subsequent observation times. In other words, the differences between using different number of iterations are expected to be more and more pronounced if we further extend the integration time. On the other hand, we can expect fewer iterations if the observation frequency is higher. Therefore, a wise way of choosing the number of iterations for different observation operators at different observation times can improve the efficiency of the PFF. Note that the PFF is based on a steepest descent algorithm for the KL divergence. There is little experience with minimization methods for pdfs, but it is highly likely that more efficient schemes are possible. We have to leave research in this direction to a future paper.

### 5.3 The prior assumption

One advantage of the PFF is that there is no assumption on the distribution of the prior, as long as we are able to derive the gradient of its logarithm analytically. In the previous section, we have assumed the prior to be Gaussian, to compare with the LETKF. However, the Gaussian assumption for the prior is definitely not optimal, for example, for hydrometeor‐related variables in atmospheric models (Posselt *et al*., 2014), or as a results of nonlinear observation operators at a previous assimilation step.

An example of a more desirable choice of the prior is a Gaussian mixture pdf. One way to construct the Gaussian mixture pdf is to first apply clustering analysis for the particles and then construct a Gaussian pdf for each cluster independently; see for example, Bengtsson *et al*. (2003). We then sum all the Gaussian pdfs with their weights proportional to the number of particles in each cluster to form the Gaussian mixture pdf. As for the way to conduct the clustering analysis, we should note that any method requiring repeated calculations of the distance between particles can be computationally prohibitive for a high‐dimensional system. For example, *k*‐means clustering can be too expensive. Other methods, such as agglomerative hierarchical clustering, in which we only need to calculate the distance between particles once, might be affordable. Although the agglomerative hierarchical clustering method seems promising, we found that it is difficult to construct the Gaussian mixture prior in the system with the square observations. The reason is in the following: despite many of the marginal distributions for the observed variables in the prior being bimodal distributed, in which case two clusters might seem to be enough, the joint distribution for all the observed variables can have many more modes. For example, we can have four modes for a two‐dimensional system if the two variables are independent and both have bimodal marginal distribution. Given that we only have 20 particles, it is difficult to construct a proper Gaussian mixture prior based on the clustering method: the number of modes is just too high.

Another possible way is to assume the prior to be a Gaussian mixture with equal weights, and with the same covariance **D** for each component. The mean of each component is set to be the state of each particle $x_{0}^{i}$, and the Gaussian mixture can be written as

(40) $$p\left(x_{0}\right)=\frac{1}{N_{p}}\sum_{i=1}^{N_{p}}N\left(x_{0}^{i},D\right)$$

In this case, the problem of determining the prior reduces to determining the covariance in each component of the Gaussian mixture. We can determine this covariance **D** by assuming that the covariance of the Gaussian mixture is equal to the sample covariance of all particles together. The covariance of the Gaussian mixture can be written as

(41) $$\begin{array}{c} \mathrm{Cov}\left(x_{0}\right)=D+\frac{1}{N_{p}}\sum_{i=1}^{N_{p}}\left(x_{0}^{i}-\bar{x_{0}}\right){\left(x_{0}^{i}-\bar{x_{0}}\right)}^{T}\\ \bar{x_{0}}=\frac{1}{N_{p}}\sum_{i=1}^{N_{p}}x_{0}^{i} \end{array}$$

where $\bar{x_{0}}$ is the ensemble mean of the prior. The sample covariance of the particles is

(42) $$B=\mathrm{Cov}\left({\left\{x_{0}^{i}\right\}}_{i=1}^{N_{p}}\right)=\frac{1}{N_{p}-1}\sum_{i=1}^{N_{p}}\left(x_{0}^{i}-\bar{x_{0}}\right){\left(x_{0}^{i}-\bar{x_{0}}\right)}^{T}$$

We can immediately obtain the covariance matrix **D** as

(43) $$D=\frac{B}{N_{p}}$$

Experiments with the Gaussian mixture in Equation (40) show that the covariance for each component **D** is too narrow, making the update of each particle very limited (not shown). We can improve this update by making **D** wider through multiplication with a scalar, so that each component is able to interact with each other. However, we note that making **D** wider is to some extent equivalent to inflating the prior. We should not make **D** so wide that the information of the prior will be lost. Nevertheless, we can still tune the width of **D** to seek an optimal width for our system. We have tried this Gaussian mixture prior in the system with square observations, but the RMSE of this ensemble does not show significant improvement over the ensemble with Gaussian prior (not shown). This might be due to the characteristics of the current system, since the relationship even between neighboring variables is generally weak in the Lorenz 1996 model. We still expect an improvement of PFF performance using the Gaussian mixture prior in a real atmospheric model over the Gaussian prior, since some nonlinear relationships, such as hydrometeor variables, will be apparent. Detailed examinations of different prior assumptions on the performance of the PFF are needed in future studies.

## 6 CONCLUSIONS AND DISCUSSION

The particle flow filter (PFF) is a recently developed Monte Carlo filter based on a deterministic flow, which naturally avoids the weight degeneracy problem and thus has potential to be applied to high‐dimensional problems. The PFF optimally transforms the particles from the prior distribution to the posterior distribution, maintaining equal weight for all the particles at all iteration steps. With the assumption that the particle flow is embedded in a reproducing kernel Hilbert space (RKHS), we are able to derive an analytical expression for the particle flow that minimizes the Kullback–Leibler divergence (KL divergence) between intermediate pdfs and the posterior pdf, starting at the prior.

The particle flow is composed of two terms: the weighted average of the gradient of the logarithm of the posterior and the divergence of the kernel. With the former term alone, the particles are driven to the mode of the posterior. This can be demonstrated when we have only one particle. In this case, the divergence of the kernel vanishes and the particle flow is equivalent to a 3DVar. The divergence of the kernel acts to repel the particles away from each other. When the summation of two terms for all the particles balance each other, the particle distribution will describe the posterior distribution.

In the limit of infinite number of particles, the solution from the PFF is independent of the choice of the kernel. However, since we have only a finite number of particles, the choice of the kernel becomes critical. When the PFF was first developed, a scalar Gaussian kernel was found to work well in a relatively low‐dimensional system with dense observations. However, we find that in the sparsely observed high‐dimensional system, in which the variance of the gradient of posterior among variables can be very large, the scalar kernel fails to maintain the variance of the marginal distribution of the observed variables. To tackle this problem, a new matrix‐valued Gaussian kernel is proposed in this study. The proposed kernel is a diagonal matrix with a different Gaussian scalar kernel in the diagonal entries. The advantage of this kernel is that it independently measures the distance between particles in each direction, ensuring that the marginal distributions will not collapse.

The PFF with the newly proposed matrix‐valued kernel is tested with a sequential data assimilation experiment in a 1,000‐dimensional Lorenz 96 system, with only 25% of the system observed every 20 time steps. With linear observations, the performance of the PFF is similar to a well‐tuned LETKF. Note that, with a proper choice of kernel width, the PFF does not require inflation of the prior, while an inflated prior is needed for the LETKF to achieve similar results. With nonlinear observations, the PFF outperforms the LETKF in terms of the RMSE in observational space and the rank histogram of the prior at observation times. We have separately examined two aspects of the nonlinear observations: a multimodal structure in the likelihood and the dependency of the linearized observation operator on the state. Since the PFF is able to evaluate the linearized observation operator locally for each particle, the PFF can capture the multimodal distribution of the likelihood in the absolute value and square observation operators, which also improves the update of the unobserved variables through a better covariance structure in the system. On the other hand, since the PFF iteratively updates the linearized observation operator, it can much better capture the nonlinear relation between model state and observations.

The sensitivity of settings in the PFF to its performance is also examined, but much more work is needed. In terms of kernel width, it is found that a wide (narrow) kernel tends to make the posterior wider (narrower), but a strong point of the methodology is that the sensitivity to the scaling factor is small with good performance over a range of a factor 10. An optimal width is found when the scaling factor is the reciprocal of the number of particles. While we explored a diagonal matrix‐valued kernel, a possible extension is to explore the off‐diagonal elements to provide a smoother repelling force. We did not need that in our experiments, but this might be useful in more realistic models. In terms of the iteration number, it is found that fewer iterations are needed for the linear observation than the nonlinear observations to reach a steady‐state posterior solution. In addition, the iterations needed at different observation times can be quite different. We use the quasi‐Newton method to accelerate the convergence, with the preconditioner chosen to be the localized background error covariance from the particles, but other methods might be more beneficial. In terms of the assumption for the prior, we propose different ways of efficiently constructing a Gaussian mixture prior. The sensitivity experiments show that the PFF with a Gaussian mixture prior does not show significant improvement over the PFF with a Gaussian prior, mainly related to the difficulty of applying an efficient clustering algorithm to define the mixtures. Furthermore, we pushed the method by only using 20 particles, in which case clustering is perhaps overdoing it. Other possibilities include a hybrid covariance between ensemble covariance and a static (climatology) covariance. This might especially be of interest when Gaussian mixtures are used with a small ensemble size.

When the PFF is applied to a real atmospheric model with complex observation operators, the adjoint of the observational operator may not always be available. In the ensemble Kalman filter, the ensemble covariance between state and observation space can be used to replace the adjoint model. However, when the observation is highly nonlinear, the ensemble covariance will not be accurate. An alternative solution to avoid the adjoint is to assume that the observational operator is embedded in a RKHS (Pulido *et al*., 2019). A similar trick is used as in the PFF, and the gradient of the observational operator can be obtained without need for the full adjoint model. Pulido *et al*., 2019 have also shown that the performance of PFF using the RKHS approximation for the linearized observation operator is better than using the ensemble covariance approximation.

Finally, the PFF that has been developed here is a filter, but it can easily be extended to a smoother. Since the PFF is computationally similar to an ensemble of 3DVars, this would be similar to an extension to an ensemble 4DVars. Indeed, as an example, the “Ensemble of data assimilations” promoted by ECMWF, which is essentially an ensemble smoother, can be transformed relatively easy into a fully nonlinear data‐assimilation system via the PFF. The main difference is that the 4DVars will have to communicate at every iteration by sending over full state vectors from one minimization to the other. One can also envisage a scheme in which communication is not at every iteration step to speed up calculations. There remains much to do before this is reality, but there is a clear path ahead.

## APPENDIX A THE DERIVATION OF THE PFF IN EQUATIONS (6) AND (7)

The Kullback–Leibler divergence (KL divergence) between the intermediate pdf at pseudo time *s* *q*_*s*_ and targeted pdf *p*(**x**|**y**) is given by Equation (A1):

(A1) $$\mathrm{KL}\left(q_{s}\right)≔\int q_{s}\left(x\right)\log\left(\frac{q_{s}\left(x\right)}{p\left(x|y\right)}\right)dx$$

The goal of this derivation is to find the appropriate flow field **f**_*s*_, such that the KL divergence decreases with pseudo time:

(A2) $$\;\frac{d}{\mathrm{ds}}x=f_{s}\left(x\right),s\in \left[0,\infty \right]$$

(A3) $$\mathrm{KL}\left(q_{s+∆s}\right)\leq \mathrm{KL}\left(q_{s}\right)$$

where *q*_*s* + ∆*s*_ is the imtermediate pdf at pseudo time *s* + ∆*s*.

To achieve the goal, we require that the derivate of KL divergence to pseudo time is negative:

(A4) $$\frac{\mathrm{dKL}}{\mathrm{ds}}\leq 0$$

That is,

(A5) $$\begin{array}{cc} \frac{\mathrm{dKL}}{\mathrm{ds}} & =\frac{d}{\mathrm{ds}}\int q_{s}\left(x\right)\log\left(\frac{q_{s}\left(x\right)}{p\left(x|y\right)}\right)dx\\ & =\int \frac{\partial }{\partial s}\left\{q_{s}\left(x\right)\log\left(\frac{q_{s}\left(x\right)}{p\left(x|y\right)}\right)\right\}dx\\ & =\int \frac{\partial q_{s}\left(x\right)}{\partial s}\left\{\log\left(\frac{q_{s}\left(x\right)}{p\left(x|y\right)}\right)+1\right\}dx\leq 0 \end{array}$$

Since we assume there is only advection in the transformation of the state **x**, the pseudo time rate change of the intermediate pdf *q*_*s*_ is characterized by the corresponding Liouville equation:

(A6) $$\frac{\partial q_{s}\left(x\right)}{\mathrm{\partial s}}+\nabla_{x}\cdot \left(f_{s}\left(x\right)q_{s}\left(x\right)\right)=0$$

Based on the Liouville equation (Equation (A6)), Equation (A5) can be written as

(A7) $$\frac{\mathrm{dKL}}{\mathrm{ds}}=-\int \nabla_{x}\cdot \left(f_{s}\left(x\right)q_{s}\left(x\right)\right)\left\{\log\left(\frac{q_{s}\left(x\right)}{p\left(x|y\right)}\right)+1\right\}dx$$

Integrating by parts, Equation (A7) can be further written as

(A8) $$\frac{\mathrm{dKL}}{\mathrm{ds}}=\int f_{s}\left(x\right)q_{s}\left(x\right)\cdot \nabla_{x}\left\{\log\left(\frac{q_{s}\left(x\right)}{p\left(x|y\right)}\right)+1\right\}dx$$

We have used the fact that *q*_*s*_ should approach zero on the boundary to assure that the integral of *q*_*s*_ in the whole domain is finite. Equation (A8) can be further written as

(A9) $$\begin{array}{cc} \frac{\mathrm{dKL}}{\mathrm{ds}} & =\int f_{s}\left(x\right)q_{s}\left(x\right)\cdot \nabla_{x}\left\{\log\left(q_{s}\left(x\right)\right)-\log\left(p\left(x|y\right)\right)\right\}dx\\ & =\int f_{s}\left(x\right)\cdot \nabla_{x}q_{s}\left(x\right)-f_{s}\left(x\right)q_{s}\left(x\right)\cdot \nabla_{x}\log\left(p\left(x|y\right)\right)dx\\ & =-\int q_{s}\left(x\right)\nabla_{x}\cdot f_{s}\left(x\right)+f_{s}\left(x\right)q_{s}\left(x\right)\cdot \nabla_{x}\log\left(p\left(x|y\right)\right)dx \end{array}$$

where the integration by parts has been used again from line two to line three in Equation (A9).

We now assume the solution of **f**_*s*_ is embedded in a reproducing kernel Hilbert space (RKHS). Every function in a RKHS can be written as

(A10) $$f_{s}\left(x\right)=\left\langle K\left(x,\cdot \right),f_{s}\left(\cdot \right)\right\rangle \in {\mathbb{R}}^{n_{x}}$$

where **K** is the matrix‐valued kernel. The bracket is the inner product in the RKHS. Based on Equation (A10), the divergence of **f**_*s*_ can be written as

(A11) $$\nabla_{x}\cdot \left[f_{s}\left(x\right)\right]=\nabla_{x}\cdot \left\langle K\left(x,\cdot \right),f_{s}\left(\cdot \right)\right\rangle ={\left\langle \nabla_{x}\cdot K\left(x,\cdot \right),f_{s}\left(\cdot \right)\right\rangle }_{1}\in \mathbb{R}$$

Based on Equations (A10) and (A11), the rate of change of the KL divergence in Equation (A9) can be written as

(A12) $$\begin{array}{c} -\int q_{s}\left(x\right){\left\langle \nabla_{x}\cdot K\left(x,\cdot \right),f_{s}\left(\cdot \right)\right\rangle }_{1}+\left\langle K\left(x,\cdot \right),f_{s}\left(\cdot \right)\right\rangle q_{s}\left(x\right)\cdot \nabla_{x}\\ \log\left(p\left(x|y\right)\right)dx\\ =-\int q_{s}\left(x\right)\{{\left\langle \nabla_{x}\cdot K\left(x,\cdot \right),f_{s}\left(\cdot \right)\right\rangle }_{1}+\langle K\left(x,\cdot \right)\nabla_{x}\\ {\log\left(p\left(x|y\right)\right),f_{s}\left(\cdot \right)\rangle }_{1}\}dx\\ =\left\langle -\int q_{s}\left(x\right)\{\nabla_{x}\cdot K\left(x,\cdot \right)+K\left(x,\cdot \right)\nabla_{x}\right.\\ {\left.\log\left(p\left(x|y\right)\right)\}dx,f_{s}\left(\cdot \right)\right\rangle }_{1} \end{array}$$

To make the pseudo time rate change of KL‐divergence negative, that is, Equation (A4), we choose **f**_*s*_ based on Equation (A12) as

(A13) $$\begin{array}{cc} f_{s}\left(\cdot \right) & =D\int q_{s}\left(x\right)\left\{\nabla_{x}\cdot K\left(x,\cdot \right)+K\left(x,\cdot \right)\nabla_{x}\log\left(p\left(x|y\right)\right)\right\}dx\\ & =DI_{f} \end{array}$$

in which **D** is a positive definite matrix included to ensure that **f**_*s*_ has the right physical dimension. With this choice, and abbreviating the integral as **I**_*f*_, Equation (A12) becomes

(A14) $$\frac{\mathrm{dKL}}{\mathrm{ds}}=\left\langle -I_{f},DI_{f}\right\rangle \leq 0$$

Here we used that **D** is positive definite with respect to the inner product. Note that **f**_*s*_ is pointing in the direction with the greatest descent of KL divergence, modified by matrix **D**.

To implement Equation (A13), we need to represent *q*_*s*_(**x**) with the particle representation. Assuming the particles are ${\left\{x_{s}^{i}\right\}}_{i=1}^{N_{p}}$, then

(A15) $$q_{s}\left(x\right)=\frac{1}{N_{p}}\sum_{i=1}^{N_{p}}\delta \left(x-x_{s}^{i}\right)$$

With Equations (A13) and (A15), we can obtain the solution of the particle flow:

(A16) $$f_{s}\left(\cdot \right)=\frac{1}{N_{p}}D\sum_{i=1}^{N_{p}}\nabla_{x_{s}^{i}}\cdot K\left(x_{s}^{i},\cdot \right)+K\left(x_{s}^{i},\cdot \right)\nabla_{x_{s}^{i}}\log\left(p\left(x_{s}^{i}|y\right)\right)$$

where

(A17) $$\nabla_{x_{s}^{i}}\cdot K\left(x_{s}^{i},\cdot \right)={\nabla_{x}\cdot K\left(x,\cdot \right)|}_{x=x_{s}^{i}}$$

and

(A18) $$\nabla_{x_{s}^{i}}\log\left(p\left(x_{s}^{i}|y\right)\right)={\nabla_{x}\log\left(p\left(x|y\right)\right)|}_{x=x_{s}^{i}}$$
